# Supplementary material for: Genome-wide association study meta-analysis identifies susceptibility loci informing Ewing sarcoma etiology and potential mechanisms of risk
Source: medRxiv. 2026 Feb 9:2026.02.06.26345779. Preprint. [Version 1] doi: 10.64898/2026.02.06.26345779 (PMC12919121; doi:10.64898/2026.02.06.26345779)
Supplement: Supplement 1 [file media-1.pdf]

Supplemental Table 1. Case Characteristics by metastatic disease and overall for EwS cases with available clinical data*.

Trait	N (%) or mean (SD)
**Age at diagnosis**	14.5 (7.6)
**Sex**	
Male	709 (55.9)
Female	559 (44.1)
**Metastasis at Diagnosis**	
Yes	268 (28.7)
No	666 (71.3)
**Tumor Location**	
Head/Neck	71 (7.9)
Chest	152 (16.9)
Spine	125 (13.9)
Upper Extremity	83 (9.2)
Abdomen	11 (1.2)
Pelvis	207 (23.0)
Lower Extremity	250 (27.8)

*Clinical data was not available for GMKF, St. Jude, CESS cases and was not available across all cases from the original GWAS.

Supplemental Table 2. Contributing Studies and case/control totals for each study and each meta-analysis set.

Analysis Set	Contributing Studies/Institutions for Cases[a]	Genotyping Array	Fixed Effects Meta-analysis[b]			METAL Meta-analysis[c]			
			Cases	Controls[a]	Total	Cases	Controls[a]	Total	ESS[d]
IC-GWAS1	Institut Curie (IC) samples from published GWAS 1	Illumina 610 Quadv1 array	401	682	1,083	401	682	1,083	1,010
Omni Set	Centers for Cancer Research (CCR), Bone Disease Study, Institut Curie (IC)	Illumina OmniExpress-24 v1.1 array	170	340	510	170	340	510	453
CCSS	Childhood Cancer Survivor Study (CCSS)	Human Omni5Exome array	159	319	478	159	319	478	424
GSA Set	Children's Oncology Group (COG), Institut Curie (IC), Michigan Blood Spots	Infinium Global Screening Array-24 v2-0	551	5,510	6,061	551	5,510	6,061	2,004
CESS	Cooperative Ewing Sarcoma Study (CESS)	Infinium Global Screening Array-24 v1-3	335	2,816	3,151	335	2,816	3,151	1,198
SJLIFE	St. Jude Lifetime Cohort Study at St. Jude Children's Research Hospital	Whole genome sequencing	111	285	396	111	285	396	320
GMKF	Gabriela Miller Kids Foundation (GMKF) Parent-Offspring study	Whole genome sequencing	-	-	-	287	573	870	765
Totals[c]			1,727	9,952	11,679	2,014	10,525	12,539	

[a]Controls were ancestry-matched and sourced from cancer-free individuals from the Prostate, Lung, Colorectal and Ovarian Screening Trial (PLCO), American Cancer Society Prevention Study II, the Spanish Bladder Cancer Study, and Study of Health In Pomerania - TREND (SHIP-TREND) study. Gabriela Miller Kids First (GMKF) controls were parents of affected EwS patients.

[b]Primary Fixed Effects Meta-analysis was retricted to 80% genetic similarity to European samples and case-control study sets.

[c]Meta-analysis in METAL was used to combine *P* values across studies taking sample size and direction of effect into account while combining family-based TDT results with case-control results.

[d] Effective sample size (ESS) used in sample weighted meta-analysis using METAL. ESS=4xcase N X control N/(case N + control N).

Supplemental Table 3. Allele frequency of the effect (risk) allele for genome wide significant lead variants in different populations.

Chr	Locus[a]	rsID[b]	Referent Allele	Effect Allele	Odds Ratio	EAF^{Global}	EAF^{EUR}	EAF^{AFR}	EAF^{ASN}	EAF^{EAS}	EAF^{SAS}	EAF^{LAT1}	EAF^{LAT2}
1	1p36.22	rs11589058	A	T	2.11	0.7574	0.7971	0.5346	0.9460	**0.9500**	0.8800	0.7050	0.8770
	1p36.13	rs545301	C	T	2.49	0.0728	0.0100	0.2651	**0.3420**	0.3330	0.1540	0.0960	0.2740
5	5q32.3	rs1488534366*	C	CT	1.36	0.8893	0.8580	0.9981	0.9900	**1.0000**	**1.0000**	**1.0000**	**1.0000**
	6p25.1	rs17142617	A	G	1.53	0.1351	0.1237	0.2651	**0.1890**	0.1840	0.0790	0.1780	0.0769
6	6p22.1	rs6935895	T	A	2.95	0.0534	0.0100	**0.2834**	0.0270	0.0300	0.0000	0.0890	0.0260
7	7q32.3	rs350653	T	C	1.95	0.8351	0.9003	0.4956	0.9820	**0.9900**	0.9100	0.8560	0.9390
8	8q24.21	rs10108830	T	A	1.34	0.3212	0.3296	0.2168	0.4720	0.4900	0.2400	0.4250	0.5380
10	10q21.2	rs10822056	C	T	1.63	0.5136	0.5183	**0.5933**	0.3523	0.2935	0.5460	0.5292	0.3370
11	11q24.1	rs10790459	T	G	1.30	0.7155	0.7226	0.4357	0.8325	0.8168	0.7370	0.6270	0.7377
12	12q14.3	rs150006321	C	T	1.31	0.7942	0.7518	**0.9538**	0.8330	0.8600	0.6800	0.7880	0.5670
14	14q13.3	rs2764962	T	C	1.26	0.5887	0.5675	**0.8154**	0.9060	0.9060	0.6138	0.6680	0.7617
15	15q15.1	rs9919974	T	G	1.67	0.2507	**0.2831**	0.1466	0.0180	0.0100	0.2000	0.1780	0.1100
18	18q21.2	rs1563410	C	T	1.25	0.4700	0.4813	0.4019	**0.7560**	0.7160	0.5820	0.4720	0.4582
20	20p11.22	rs6047482	T	A	1.71	0.6683	**0.7288**	0.3487	0.6880	0.6900	0.6800	0.6510	0.6460

EAF = Effect Allele Frequency , EUR=European, AFR=African, ASN=Asian, EAS=East Asian, SAS=South Asian, LAT1=Latin American 1, LAT2=Latin American 2.

EAF is based on the ALFA project which provides aggregate allele frequency from dbGaP.

*EAF based on lead variant from METAL as lead variant in case-control meta-analysis (indel chr5:146327889) was not listed in dbSNP.

Supplemental Table 4. Study-specific summary statistics for EwS meta-analysis. Variants were not included if minor allele frequency was < 1%. Study estimates were calculated in Plink for all studies except CESS which used SNPTEST.

				Institut Curie Set 1 - 1st Published GWAS (N=1,083)					
Chr	Position[a]	rsID[b]	Risk allele	OR	SE	Lower 95% CI	Upper 95% CI	*P* value	INFO[c]
1	10,987,244	rs11589058	T	2.46	0.146	1.85	3.27	5.94E-10	0.95
1	17,873,309	**rs545301**	T	1.86	0.471	0.74	4.68	0.1875	0.77
5	146,327,889	**chr5:146327889**	CT	1.35	0.116	1.07	1.69	0.0103	0.88
6	6,837,792	rs17142617	G	1.76	0.126	1.38	2.25	6.85E-06	0.88
6	29,543,984	**rs6935895**	A	2.95	0.470	1.18	7.36	0.0200	0.97
7	131,030,999	**rs350653**	C	1.77	0.206	1.18	2.64	0.0058	0.84
8	129,448,673	**rs10108830**	A	1.32	0.092	1.10	1.58	0.0027	0.98
10	62,754,351	rs10822056	T	1.93	0.100	1.59	2.35	4.69E-11	0.99
11	121,767,808	rs10790456	G	1.21	0.102	0.99	1.48	0.0614	0.80
12	66,069,074	**rs150006321**	T	1.33	0.110	1.07	1.65	0.0092	0.95
14	36,759,015	**rs2764962**	C	1.19	0.094	0.99	1.44	0.0600	0.92
15	40,045,261	rs9919974	G	1.61	0.098	1.33	1.95	1.09E-06	0.98
18	51,335,382	**rs1563410**	T	1.19	0.090	1.00	1.42	0.0549	0.98
20	21,559,045	rs6047482	A	1.81	0.117	1.44	2.27	3.78E-07	0.95

	Omni: Bone Disease Study, Institut Curie Set # 2, Centers for Cancer Research (N=510)						Childhood Cancer Survivor Study (N=478)					
rsID[b]	OR	SE	Lower 95% CI	Upper 95% CI	*P* value	INFO[c]	OR	SE	Lower 95% CI	Upper 95% CI	*P* value	INFO[c]
rs11589058	2.05	0.184	1.43	2.94	9.49E-05	0.82	2.00	0.212	1.32	3.02	0.0011	0.99
**rs545301**	NA	NA	NA	NA	NA	0.77	1.71	0.693	0.44	6.64	0.4393	1.00
**chr5:146327889**	1.84	0.188	1.27	2.66	0.0012	0.91	1.64	0.183	1.15	2.35	0.0068	0.97
rs17142617	1.22	0.195	0.83	1.79	0.3129	0.97	1.97	0.195	1.35	2.89	0.0005	1.00
**rs6935895**	NA	NA	NA	NA	NA	1.00	1.06	0.654	0.29	3.82	0.9283	1.00
**rs350653**	2.04	0.293	1.15	3.62	0.0152	0.85	1.08	0.268	0.64	1.83	0.7773	0.98
**rs10108830**	1.26	0.142	0.95	1.66	0.1070	0.98	1.33	0.141	1.01	1.75	0.0414	1.00
rs10822056	1.66	0.148	1.24	2.22	0.0007	0.97	1.72	0.147	1.29	2.29	0.0002	1.00
rs10790456	1.33	0.152	0.99	1.80	0.0593	1.00	1.43	0.173	1.02	2.00	0.0389	1.00
**rs150006321**	1.24	0.168	0.89	1.73	0.1993	0.94	1.42	0.171	1.02	1.99	0.0390	1.00
**rs2764962**	1.21	0.140	0.92	1.59	0.1804	1.00	1.30	0.142	0.98	1.71	0.0664	1.00
rs9919974	1.69	0.144	1.28	2.24	0.0003	0.92	1.64	0.146	1.23	2.18	0.0007	0.99
**rs1563410**	1.39	0.139	1.06	1.82	0.0185	0.99	1.26	0.147	0.95	1.68	0.1121	1.00
rs6047482	1.63	0.171	1.17	2.28	0.0042	0.96	1.86	0.181	1.31	2.65	0.0006	1.00

	GSA: Children's Oncology Group, Michigan Blood Spots, Institut Curie Set # 3 (N=6,061)						CESS (N=3,151)					
rsID[b]	OR	SE	Lower 95% CI	Upper 95% CI	*P* value	INFO[c]	OR	SE	Lower 95% CI	Upper 95% CI	*P* value	INFO[c]
rs11589058	1.95	0.098	1.61	2.37	7.93E-12	0.98	1.94	0.154	1.43	2.63	1.51E-06	0.96
**rs545301**	2.56	0.176	1.81	3.61	1.03E-07	0.94	3.67	0.559	1.23	10.98	0.7915	0.99
**chr5:146327889**	1.29	0.082	1.10	1.52	1.70E-03	0.91	1.09	0.208	0.95	1.25	0.7161	0.92
rs17142617	1.40	0.085	1.18	1.65	8.34E-05	1.00	1.53	0.139	1.16	2.00	0.0825	0.93
**rs6935895**	3.13	0.158	2.30	4.26	4.46E-13	0.98	NA	NA	NA	NA	NA	NA
**rs350653**	2.25	0.150	1.67	3.01	4.49E-08	0.98	2.32	0.229	1.48	3.63	1.29E-05	0.96
**rs10108830**	1.31	0.065	1.15	1.48	4.36E-05	0.99	1.50	0.104	1.22	1.84	0.0034	0.98
rs10822056	1.55	0.066	1.36	1.77	3.28E-11	0.99	1.51	0.105	1.23	1.85	1.18E-05	0.96
rs10790456	1.28	0.074	1.10	1.48	9.71E-04	0.94	1.22	0.096	1.01	1.48	0.0673	0.92
**rs150006321**	1.31	0.077	1.13	1.53	4.04E-04	0.99	1.25	0.094	1.04	1.50	0.6678	0.96
**rs2764962**	1.34	0.067	1.18	1.53	1.07E-05	1.00	1.23	0.102	1.01	1.50	0.0321	1.00
rs9919974	1.70	0.066	1.49	1.93	9.88E-16	0.99	1.67	0.106	1.35	2.05	3.66E-08	0.99
**rs1563410**	1.31	0.06	1.16	1.48	2.10E-05	0.99	1.18	0.100	0.97	1.44	0.7050	0.96
rs6047482	1.72	0.083	1.46	2.02	6.91E-11	0.99	1.74	0.132	1.34	2.25	2.97E-05	0.95

	St. Jude Life Study (N=396)				
rsID[b]	OR	SE	Lower 95% CI	Upper 95% CI	*P* value
rs11589058	3.45	0.2793	2.00	5.97	######
**rs545301**	NA	NA	NA	NA	NA
**chr5:146327889**	NA	NA	NA	NA	NA
rs17142617	1.82	0.2345	1.15	2.88	0.0107
**rs6935895**	NA	NA	NA	NA	NA
**rs350653**	NA	NA	NA	NA	NA
**rs10108830**	NA	NA	NA	NA	NA
rs10822056	1.60	0.177	1.13	2.27	0.0078
rs10790456	1.82	0.204	0.87	1.93	0.0033
**rs150006321**	NA	NA	NA	NA	NA
**rs2764962**	1.12	0.159	0.82	1.53	0.4799
rs9919974	NA	NA	NA	NA	NA
**rs1563410**	1.09	0.163	0.79	1.50	0.6043
rs6047482	1.35	0.196	0.92	1.98	0.1302

	Gabriela Miller Kids First EwS Trios[d] (N=860)						
rsID[b]	T	U	TDT OR	CHISQ	Lower 95% CI	Upper 95% CI	*P* value
rs11589058	48	25	1.92	7.25	1.19	3.09	0.0071
**rs545301**	9	1	9.00	6.40	1.64	49.38	0.0114
**chr5:146327889**	69	68	1.02	0.01	0.72	1.43	0.9319
rs17142617	40	15	2.67	11.36	1.51	4.72	7.49E-04
**rs6935895**	1	0	N/A	1.00	N/A	N/A	0.3173
**rs350653**	16	8	2.00	2.67	0.87	4.59	0.1025
**rs10108830**	123	89	1.38	5.45	1.05	1.81	0.0195
rs10822056	167	70	2.39	39.70	1.82	3.13	2.96E-10
rs10790456	114	71	1.61	10.00	1.19	2.16	0.0016
**rs150006321**	34	24	1.42	1.72	0.84	2.39	0.1892
**rs2764962**	77	78	0.99	0.01	0.72	1.35	0.936
rs9919974	111	49	2.65	24.02	1.79	3.92	9.51E-07
**rs1563410**	135	93	1.45	7.74	1.12	1.88	0.0054
rs6047482	37	35	1.05	0.06	0.70	1.57	0.8137

[a]SNP position according to NCBI Human Genome Build 38.

[b]Variants in bold face were newly discovered.

[c]Imputation information score. No Info score listed for SJLIFE or GMKF because data was whole genome sequencing, not imputation.

[d]OR and test statistics are based on Transmission Disequilibrium Test (TDT). T and U represent numbers of transmitted and untransmitted alleles respectively.

Supplemental Table 5. Leave one out (LOO) fixed effects (FE) meta-analysis odds ratio (OR) and 95% confidence intervals (CI). FE meta-analysis was conducted after leaving out each study one at a time.

		Removing GSA				Removing IC-GWAS1				Removing Omni Set				Removing CCSS Set				Removing CESS set				Removing SJLIFE set			
**Chr**	**Variant[a]**	**OR**	**Lower 95% CI**	**Upper 95% CI**	***P* value**	**OR**	**Lower 95% CI**	**Upper 95% CI**	***P* value**	**OR**	**Lower 95% CI**	**Upper 95% CI**	***P* value**	**OR**	**Lower 95% CI**	**Upper 95% CI**	***P* value**	**OR**	**Lower 95% CI**	**Upper 95% CI**	***P* value**	**OR**	**Lower 95% CI**	**Upper 95% CI**	***P* value**
1	rs11589058	2.22	1.65	3.43	3.78E-23	2.04	1.60	2.81	4.51E-25	2.12	1.66	2.92	7.82E-30	2.12	1.67	2.91	7.72E-31	2.15	1.67	3.00	3.31E-29	2.06	1.64	2.77	1.42E-29
	**rs545301**	2.28	1.22	4.27	9.80E-03	2.58	1.87	3.55	6.84E-09	2.49	1.84	3.37	3.49E-09	2.54	1.86	3.46	4.05E-09	2.41	1.76	3.30	4.35E-08	2.49	1.84	3.37	3.49E-09
5	**chr5:146327889**	1.44	1.23	1.68	6.82E-06	1.37	1.20	1.56	2.60E-10	1.32	1.18	1.49	3.38E-06	1.34	1.19	1.50	1.54E-06	1.39	1.24	1.56	3.57E-08	1.36	1.22	1.53	6.25E-08
6	rs17142617	1.64	1.42	1.89	1.79E-11	1.48	1.31	1.67	2.60E-10	1.56	1.39	1.75	1.51E-14	1.50	1.33	1.67	3.64E-12	1.53	1.36	1.72	2.12E-12	1.51	1.35	1.69	3.89E-13
	**rs6935895**	2.09	0.99	4.40	5.22E-02	1.40	1.24	1.58	1.63E-12	2.95	2.18	3.98	1.06E-13	3.11	2.32	4.17	2.87E-14	2.95	2.22	3.92	1.06E-13	2.95	2.22	3.92	1.06E-13
7	**rs350653**	1.77	1.39	2.24	2.66E-06	2.00	1.62	2.45	5.33E-11	1.94	1.59	2.35	2.53E-11	2.11	1.74	2.57	7.98E-14	1.88	1.54	2.30	8.72E-10	1.95	1.62	2.34	1.27E-12
8	**rs10108830**	1.36	1.22	1.52	5.50E-08	1.34	1.22	1.48	1.16E-09	1.35	1.23	1.47	3.94E-11	1.34	1.22	1.46	9.78E-11	1.31	1.19	1.43	1.25E-08	1.34	1.23	1.45	1.17E-11
10	rs10822056	1.70	1.52	1.89	8.10E-21	1.57	1.43	1.73	1.69E-21	1.63	1.49	1.78	1.08E-27	1.63	1.49	1.78	2.75E-27	1.66	1.51	1.82	4.38E-27	1.64	1.50	1.78	1.10E-28
11	**rs10790456**	1.31	1.16	1.48	1.43E-05	1.32	1.19	1.47	2.32E-07	1.29	1.17	1.42	3.17E-07	1.29	1.17	1.42	3.90E-07	1.31	1.18	1.45	1.79E-07	1.27	1.15	1.40	9.76E-07
12	**rs150006321**	1.30	1.14	1.48	6.36E-05	1.30	1.16	1.45	3.11E-06	1.31	1.19	1.46	2.09E-07	1.30	1.17	1.44	7.39E-07	1.32	1.18	1.47	6.17E-07	1.31	1.18	1.44	9.39E-08
14	**rs2764962**	1.21	1.09	1.34	3.51E-04	1.28	1.17	1.40	1.51E-07	1.27	1.16	1.38	7.14E-08	1.26	1.15	1.37	1.67E-07	1.27	1.16	1.39	2.40E-07	1.27	1.17	1.38	2.83E-08
15	rs9919974	1.65	1.47	1.85	2.63E-17	1.68	1.53	1.85	3.06E-26	1.67	1.52	1.82	1.73E-28	1.67	1.53	1.83	6.90E-29	1.67	1.52	1.83	2.34E-26	1.67	1.53	1.82	2.09E-31
18	**rs1563410**	1.21	1.09	1.34	2.86E-04	1.27	1.16	1.38	1.93E-07	1.24	1.14	1.35	4.56E-07	1.25	1.15	1.36	1.30E-07	1.26	1.16	1.38	1.17E-07	1.26	1.16	1.37	2.72E-08
20	rs6047482	1.71	1.50	1.95	1.60E-15	1.69	1.51	1.89	2.88E-19	1.72	1.54	1.92	4.34E-23	1.70	1.53	1.89	2.50E-22	1.71	1.53	1.91	4.74E-21	1.74	1.57	1.94	1.01E-24

[a]Variants in bold face were newly discovered.

Supplemental Table 6. Case-trio analysis lead variant in TDT results at each significant locus from meta-analysis results. Case-parent trios were from Gabriela Miller Kid's Foundation (GMKF) set of 287 affected cases and 573 parents.

Chr	Locus[a]	rsID[b]	Position[c]	Referent Allele	Risk Allele	U	T	OR[d]	CHISQ	Association *P* value	Distance in kb
1	1p36.22	**rs879806904**	10943077	G	T	11	65	5.91	38.37	5.86E-10	44.17
	1p36.13	rs594534	17897619	C	T	5	40	8.00	27.22	1.81E-07	24.31
5	5q32.3	**rs1488534366**	145999736	A	C	9	73	8.11	49.95	1.58E-12	328.15
6	6p25.1	rs11243125	6869665	G	T	52	105	2.02	17.89	2.34E-05	31.87
	6p22.1	rs6935895	29543124	C	T	71	140	1.97	22.56	2.03E-06	0.86
7	7q32.3	**rs971539419**	131109624	C	A	5	51	10.19	37.79	7.90E-10	78.63
8	8q24.21	rs1172924357	129364956	T	G	0	29	-	29	7.24E-08	83.72
10	10q21.2	**rs937223645**	62878036	C	A	14	89	6.36	54.61	1.47E-13	123.67
11	11q24.1	**rs1307551815**	121785466	T	A	4	50	12.50	39.19	3.86E-10	17.66
12	12q14.3	**rs376370554**	66069186	A	G	3	46	15.33	37.73	8.11E-10	0.11
14	14q13.3	rs1749944	36757328	C	T	68	33	2.06	12.13	4.97E-04	1.69
15	15q15.1	**rs147622877**	40077773	T	G	7	56	8.00	38.11	6.68E-10	32.51
18	18q21.2	**rs796151360**	51039505	C	A	44	132	3.00	44	3.28E-11	295.88
20	20p11.22	**rs1202095641**	21444365	G	T	7	63	9.00	44.8	2.18E-11	114.68

[a]Cytogenetic regions according to NCBI Human Genome Build 38.

[b]Variants in bold face were signficant at genome-wide level in TDT analysis.

[c]Variant position according to NCBI Human Genome Build 38.

[d]Odds Ratio (OR) is derived from transmission disequilibrium test (TDT) and effectively the ratio between Transmitted (T) and Untransmitted (U) alleles.

Supplemental Table 7. Conditional significant variants in genome-wide significant loci for subjects >80% genetic similarity to European ancestry for Ewing sarcoma susceptibility. GWAS was conducted at a 1% minor allele threshold.

Chr	Locus[a]	rsID[b]	Position[c]	Reference Allele	Risk Allele	EAF	Marginal effect (Beta)	Marginal SE	Marginal *P* value	Index rsID[d]	Conditional effect (Beta)	Conditional SE	Conditional *P* value	Distance in Kb	LD (r) in Eur
6	6p25.1	rs367699968	6845462	T	TAC	0.00021283	-0.443363	0.0724522	9.39E-10	rs17142617	-0.4437	0.0724525	9.10E-10	7.67	0.011318
7	7q32.3	rs6978148	130993395	C	T	0.337262	-0.265796	0.0448301	3.05E-09	rs350653	-0.2954	0.0450676	5.55E-11	37.60	-0.11276
15	15q15.1	rs41500744	40086829	C	A	0.186815	-0.692191	0.0649463	1.60E-26	rs9919974	-0.5578	0.0668998	7.53E-17	41.57	-0.26904
20	20p11.23	rs6035886	21555002	C	T	0.0977706	0.472024	0.0591816	1.51E-15	rs6047482	0.3908	0.059955	7.12E-11	4.04	-0.19577

[a]Cytogenetic regions according to NCBI Human Genome Build 38.

[b]Variants identified as independent .

[c]Variant position according to NCBI Human Genome Build 38.

[d]Lead variant in GWAS conditioned upon in the model.

Supplemental Table 8. Contributing samples for case-only GWAS by sex, metastasis, and age at diagnosis. Case-only analysis was restricted to individuals with > 80% genetic similarlity to European reference populations.

Analysis Set	Contributing Studies/Institutions for Cases*	Sex N(%)		Metastasis N (%)		Age at Diagnosis N (%)		Age at Diagnosis N (%)		Age at Diagnosis N (%)		Total*
		**Males**	**Females**	**Yes**	**No**	**<10**	**10+**	**<16**	**16+**	**<20**	**20+**	
GSA Set	Children's Oncology Group (COG), Institut Curie, Michigan Blood Spots	305 (55.4)	246 (44.6)	112 (25.3)	331 (74.7)	126 (25.1)	376 (74.1)	267 (60.4)	175 (39.6)	392 (88.7)	50 (11.3)	551
IC-GWAS1	Institut Curie samples from published GWAS 1	247 (61.9)	152 (38.1)	129 (34.7)	242 (65.3)	77 (20.1)	307 (79.9)	223 (58.1)	161 (41.9)	297 (77.3)	87 (22.7)	401
Omni Set	Centers for Cancer Research, Bone Disease Study, Institut Curie	84 (52.8)	75 (47.2)	27 (22.5)	93 (77.5)	20 (16.8)	99 (83.2)	65 (54.6)	54 (45.4)	87 (73.1)	32 (26.9)	170
CCSS	Childhood Cancer Survivor Study	73 (45.9)	86 (54.1)	NA	NA	49 (30.8)	110 (69.2)	116 (73.0)	43 (27.0)	151 (95.0)	8 (5.0)	159
**Total**		**709 (55.9)**	**559 (44.1)**	**268 (28.7)**	**666 (71.3)**	**272 (23.4)**	**892 (76.6)**	**671 (60.8)**	**433 (39.2)**	**927 (84.0)**	**177 (16.0)**	**1281**

*Where phenotype data was unavailable, not all available cases were included and may not sum to total.

Supplemental Table 9. Comparing statistical enrichment to consecutive GGAA repeats or FLI1 binding. 10,000 variants from the GWAS meta-analysis were selected with replacement on each chromosome, distance between those variants and GGAA repeats or FLI1 binding was selected to make a distribution to compare against lead variants.

Chromosome	rsID	Position[a]	GGAA Repeats						FLI1 binding					
			4 or more GGAA Repeats			13 or more GGAA repeats			30 or more			45 or more		
			Position nearest	Distance to	Percentile[b]	Position nearest	Distance to	Percentile[b]	Position nearest	Distance to nearest	Percentile[b]	Position nearest	Distance to nearest	Percentile[b]
1	rs11589058	10,987,244	10,987,423	179	**0.0017**	10,992,133	4,889	**0.0065**	10,987,929	685	**0.0045**	10,987,951	707	**0.0026**
	rs545301	17,873,309	17,897,593	24,284	0.1953	17,716,773	180,820	0.1926	18,359,405	486,096	0.7937	18,359,469	486,160	0.6473
5	chr5:146327889:C:CT	146,327,889	146,364,395	36,506	0.2203	146,002,535	325,354	0.2250	146,364,182	36,293	0.1471	146,364,258	36,369	0.1024
6	rs17142617	6,837,792	6,837,048	744	**0.0084**	6,023,216	814,576	0.2572	6,837,124	668	**0.0050**	6,837,029	763	**0.0028**
	rs6935895	29,543,984	29,642,448	98,464	0.5585	28,707,629	836,355	0.5034	29,964,995	421,011	0.8020	30,682,721	1,138,737	0.9186
7	rs350653	131,030,999	131,035,309	4,310	**0.0422**	131,035,408	4,409	**0.0056**	131,035,084	4,085	**0.0209**	131,035,130	4,131	**0.0129**
8	rs10108830	129,448,673	129,449,337	704	**0.0071**	130,456,762	1,008,089	0.6354	129,449,094	421	**0.0030**	129,449,161	488	**0.0023**
10	rs10822056	62,754,351	62,755,043	692	**0.0065**	62,729,283	25,068	**0.0286**	62,693,443	60,908	0.2570	62,693,310	61,041	0.1909
11	rs10790459	121,767,808	121,780,992	13,184	0.0958	121,780,992	13184	**0.0193**	121,780,889	13,081	0.0572	121,780,911	13,103	**0.0420**
12	rs150006321	66,069,074	66,067,134	1,940	**0.0166**	66,067,134	1,940	**0.0021**	66,067,464	1,610	**0.0131**	66,067,413	1,661	**0.0094**
14	rs2764962	36,759,015	36,760,335	1,320	**0.0135**	36,760,335	1,320	**0.0021**	36,760,150	1,135	**0.0078**	36,760,189	1,174	**0.0046**
15	rs9919974	40,045,261	40,077,321	4,310	0.2015	38,795,337	1,249,924	0.6525	40,046,730	1,469	**0.0090**	40,046,812	1,551	**0.0057**
18	rs1563410	51,335,382	51,327,238	8,144	0.0645	50,959,150	376,232	0.3362	51,330,383	4,999	**0.0287**	51,327,467	7,915	**0.0293**
20	rs6047482	21,559,045	21,555,863	3,182	**0.0314**	21,576,284	17,239	**0.0239**	21,556,092	2,953	**0.0168**	21,576,052	17,007	0.0523

[a]Genomic position based on NCBI Human Genome Build 38.

[b]Proportion of values less than or equal to distance of lead variant to at least 4 or more GGAA repeats, 13 or more GGAA repeats, 30 or more FLI1 binding and 45 or more FLI1 binding.

Bold-face indicates evidence of statistical enrichment for shorter distance to GGAA repeats or FLI1 binding compared to the rest of the chromosome.

GGAA repeats and FLI1 binding on the A-673 cell line are based on publicly available data from the Ewing Sarcoma Cell Line Atlas (ESCLA).

Supplemental Table 10. SNPs in newly discovered loci were evaluated in patient samples on Affymetrix and RNA-Seq technologies for their effect on gene expression. Effects (Beta), effect direction, and *P* values for the association between the risk allele in the GWAS results and expression quantitative trait loci (eQTLs) in Affymetrix and RNA-seq chips are displayed. There were 28 samples in common across both methods. Results display most significant (in Affymetrix set) SNP-eQTL association per each gene and only results with nominal $P < 0.05$ in one or both methods were listed. Bold-face indicates results under Bonferroni-corrected threshold (0.05/209 genes, $P < 2.39\times10^{-4}$).

Locus[a]	rsID	Position[a]	Referent Allele	Risk Allele	EAF[b]	Ewing GWAS Meta-analysis Result						Gene	eQTL Analysis Result: Affymetrix microarray (N=113)			eQTL Analysis Result: RNA-Seq (N=48)		
						# Sets[c]	Beta	SE	OR	*P* value	Q		Beta	Direction	*P* value	Beta	Direction	*P* value
1p36.22	rs9430161	10986798	T	G	0.7957	6	0.7420	0.0619	2.10	4.22E-33	0.5413	CASZ1	-0.3864	**DOWN**	**6.41E-06**	-0.4814	DOWN	0.0640
1p36.22	rs11576658	10977679	T	C	0.7784	6	0.5147	0.0558	1.67	2.74E-20	0.7886	TARDBP	-0.4911	**DOWN**	**1.81E-04**	-0.3525	DOWN	0.0210
1p36.22	rs2003046	10972770	A	C	0.7754	6	0.4744	0.0545	1.61	3.20E-18	0.7738	ANGPTL7	-0.2806	DOWN	5.11E-04	0.0534	UP	0.5700
1p36.22	rs9430161	10986798	T	G	0.7957	6	0.7420	0.0619	2.10	4.22E-33	0.5413	PEX14	-0.1188	DOWN	0.0293	-0.1176	DOWN	0.2500
1p36.22	rs9430161	10986798	T	G	0.7957	6	0.7420	0.0619	2.10	4.22E-33	0.5413	C1orf127	-0.0411	DOWN	0.0527	-0.3337	DOWN	0.0410
1p36.22	rs2003046	10972770	A	C	0.7754	6	0.4744	0.0545	1.61	3.20E-18	0.7738	AGTRAP	0.1486	UP	0.1550	0.3282	UP	0.0320
5q32.3	rs10988	146120433	T	C	0.7229	6	0.2334	0.04995	1.26	2.96E-06	0.73367	RBM27	-1.2839	**DOWN**	**4.23E-20**	-1.0581	**DOWN**	**1.30E-05**
5q32.3	rs2063002	146188314	G	A	0.7179	6	0.2449	0.05004	1.28	9.87E-07	0.51881	PLAC8L1	-0.6480	**DOWN**	**7.05E-06**	-0.1896	DOWN	0.25
5q32.3	rs13173462	146242909	C	T	0.7338	6	0.2675	0.05143	1.31	1.97E-07	0.61253	TCERG1	-0.2654	DOWN	0.0051	-0.1859	DOWN	0.3
5q32.3	rs13173462	146242909	C	T	0.7338	6	0.2675	0.05143	1.31	1.97E-07	0.61253	POU4F3	-0.0303	DOWN	0.2664	-0.3987	DOWN	9.20E-04
5q32.3	rs2063002	146188314	G	A	0.7179	6	0.2449	0.05004	1.28	9.87E-07	0.51881	STK32A	NA	NA	NA	-0.0834	DOWN	0.011
6p25.1	rs1286037	6843500	A	G	0.2543	6	0.2794	0.0446	1.32	3.76E-10	0.2077	RREB1	0.1365	**UP**	**4.23E-05**	0.1879	UP	0.0580
6p25.1	rs1286037	6843500	A	G	0.2543	6	0.2794	0.0446	1.32	3.76E-10	0.2077	DSP	1.1097	UP	0.0042	0.0084	UP	0.9700
6p25.1	rs1286037	6843500	A	G	0.2543	6	0.2794	0.0446	1.32	3.76E-10	0.2077	NRN1	0.1399	UP	0.6711	-0.7914	DOWN	0.0400
7q32.3	rs350655	131028942	A	C	0.9031	5	0.6525	0.0922	1.92	1.49E-12	0.1517	CPA4	-0.0360	DOWN	0.0018	0.0452	UP	0.7200
7q32.3	rs6978148	130993395	C	T	0.6520	6	0.2658	0.0448	1.30	3.05E-09	0.5177	MKLN1	0.0981	UP	0.0211	0.1125	UP	0.1700
7q32.3	rs765965	131019616	C	A	0.8562	6	0.3663	0.0665	1.44	3.60E-08	0.2346	KLF14	-0.0750	DOWN	0.0210	NA	NA	NA
7q32.3	rs6978148	130993395	C	T	0.6520	6	0.2658	0.0448	1.30	3.05E-09	0.5177	CEP41	0.1979	UP	0.0378	0.1503	UP	0.0880
7q32.3	rs6978148	130993395	C	T	0.6520	6	0.2658	0.0448	1.30	3.05E-09	0.5177	KLHDC10	0.1605	UP	0.0381	0.1225	UP	0.1900
7q32.3	rs765965	131019616	C	A	0.8562	6	0.3663	0.0665	1.44	3.60E-08	0.2346	CPA5	0.0391	UP	0.1500	-0.1164	DOWN	0.0033
7q32.3	rs6978148	130993395	C	T	0.6520	6	0.2658	0.0448	1.30	3.05E-09	0.5177	TSGA13	0.0016	UP	0.7512	0.0084	UP	0.0190
7q32.3	rs350655	131028942	A	C	0.9031	5	0.6525	0.0922	1.92	1.49E-12	0.1517	PODXL	-0.0933	DOWN	0.8174	-1.0792	DOWN	0.0170
8q24.21	rs1897447	129449239	A	G	0.3564	6	0.2753	0.0414	1.32	2.98E-11	0.5009	MYC	0.2323	UP	0.0461	0.1687	UP	0.3900
10q21.2	rs7915131	62658896	T	C	0.4402	6	0.3111	0.0411	1.36	3.99E-14	0.6324	ADO	0.4707	**UP**	**9.60E-05**	0.3090	UP	0.0450
10q21.2	rs1848797	62793174	A	G	0.3871	6	0.4030	0.0420	1.50	8.55E-22	0.2044	EGR2	1.0298	UP	0.0022	0.8437	UP	0.0240
10q21.2	rs10995250	62637161	A	G	0.6555	6	0.3033	0.0439	1.35	4.85E-12	0.9344	RTKN2	-0.3370	DOWN	0.0047	0.0160	UP	0.8900
10q21.2	rs224307	62841675	A	G	0.5950	6	0.3960	0.0439	1.49	1.82E-19	0.1879	NRBF2	0.1852	UP	0.0069	-0.0100	DOWN	0.9000
10q21.2	rs7915131	62658896	T	C	0.4402	6	0.3111	0.0411	1.36	3.99E-14	0.6324	JMJD1C-AS1	-0.0313	DOWN	0.4182	-0.0892	DOWN	0.0360
10q21.2	rs10995239	62628605	G	A	0.6555	6	0.3025	0.0438	1.35	5.02E-12	0.9283	REEP3	-0.0278	DOWN	0.5397	-0.4821	DOWN	0.0130
11q24.1	rs10790459	121767808	T	G	0.7226	6	0.2589	0.0475	1.30	5.14E-08	0.57485	SC5D	0.0347	UP	0.0358	-0.0387	DOWN	0.7800
11q24.1	rs10790459	121767808	T	G	0.7226	6	0.2589	0.0475	1.30	5.14E-08	0.57485	MIR100HG	0.6519	UP	0.0437	0.6188	UP	0.0680
11q24.1	rs7115520	121733948	C	T	0.7396	5	0.2016	0.0496	1.22	4.76E-05	0.77641	SORL1	0.4479	UP	0.0484	-0.1137	DOWN	0.3300
11q24.1	rs7937934	121764503	C	T	0.7407	5	0.2001	0.0492	1.22	4.79E-05	0.89012	SORL1	0.4479	UP	0.0484	-0.1137	DOWN	0.3300
12q14.3	rs10735933	66066014	G	A	0.6549	6	0.2117	0.0442	1.24	1.66E-06	0.59345	TMBIM4	0.1632	UP	0.0080	0.0565	UP	0.4800
12q14.3	rs1168745	66163180	G	A	0.7295	6	0.2268	0.0476	1.25	1.90E-06	0.32202	MSRB3	0.1288	UP	0.5215	0.5704	UP	0.0490
12q14.3	rs1168745	66163180	G	A	0.7295	6	0.2268	0.0476	1.25	1.90E-06	0.32202	HMGA2	0.0732	UP	0.0194	0.1164	UP	0.7200
14q13.3	rs2764962	36759015	T	C	0.5675	6	0.2314	0.0418	1.26	3.00E-08	0.8518	PAX9	-0.9001	**DOWN**	**8.85E-06**	-0.7743	DOWN	0.0220
14q13.3	rs2764962	36759015	T	C	0.5675	6	0.2314	0.0418	1.26	3.00E-08	0.8518	MIPOL1	-0.3510	**DOWN**	**9.78E-06**	-0.2702	DOWN	0.0330
14q13.3	rs2764962	36759015	T	C	0.5675	6	0.2314	0.0418	1.26	3.00E-08	0.8518	SLC25A21	-0.7701	**DOWN**	**4.22E-05**	-0.4845	DOWN	0.0015
14q13.3	rs2764962	36759015	T	C	0.5675	6	0.2314	0.0418	1.26	3.00E-08	0.8518	SLC25A21-AS1	-0.2954	DOWN	7.80E-04	-0.4752	**DOWN**	**8.30E-05**
14q13.3	rs848092	36799592	G	A	0.3405	6	0.2320	0.0419	1.26	3.12E-08	0.5936	BRMS1L	-0.1159	DOWN	0.0172	-0.0941	DOWN	0.2500
14q13.3	rs2764962	36759015	T	C	0.5675	6	0.2314	0.0418	1.26	3.00E-08	0.8518	NKX2-8	-0.0037	DOWN	0.0937	-0.0602	DOWN	0.0026
15q15.1	rs8026641	40046660	A	G	0.2774	6	0.4879	0.0426	1.63	2.28E-30	0.3138	SRP14	-0.1444	**DOWN**	**2.00E-04**	-0.1663	DOWN	0.0400
15q15.1	rs652196	40083946	A	G	0.7534	6	0.4596	0.0529	1.58	3.75E-18	0.8293	C15orf56	-0.2428	DOWN	3.22E-04	NA	NA	NA
15q15.1	rs8026641	40046660	A	G	0.2774	6	0.4879	0.0426	1.63	2.28E-30	0.3138	BMF	-0.6036	DOWN	3.60E-04	-0.7591	DOWN	0.0036
15q15.1	rs12913170	40084007	T	C	0.8280	6	0.6827	0.0672	1.98	2.78E-24	0.0780	CHST14	-0.3736	DOWN	0.0038	-0.1819	DOWN	0.1900
15q15.1	rs8026641	40046660	A	G	0.2774	6	0.4879	0.0426	1.63	2.28E-30	0.3138	CHAC1	0.1329	UP	0.0095	0.2700	UP	0.0250
15q15.1	rs8042947	40033659	G	A	0.7233	6	0.2640	0.0480	1.30	3.71E-08	0.4360	THBS1	-0.5399	DOWN	0.0220	-0.2853	DOWN	0.5000
15q15.1	rs17722526	40028066	A	G	0.3494	6	0.3836	0.0414	1.47	1.94E-20	0.1115	VPS18	0.0421	UP	0.0282	0.1243	UP	0.1600
15q15.1	rs12913170	40084007	T	C	0.8280	6	0.6827	0.0672	1.98	2.78E-24	0.0780	PAK6	-0.4905	DOWN	0.0316	NA	NA	NA
15q15.1	rs12164905	40008490	T	G	0.8234	6	0.3398	0.0590	1.40	8.24E-09	0.0500	KNSTRN	0.3715	UP	0.0462	0.3185	UP	0.1600
15q15.1	rs937213	40029923	T	C	0.4212	6	0.3089	0.0411	1.36	5.47E-14	0.4473	DISP2	0.2908	UP	0.0479	0.2294	UP	0.1100
15q15.1	rs8026641	40046660	A	G	0.2774	6	0.4879	0.0426	1.63	2.28E-30	0.3138	PPP1R14D	0.0402	UP	0.0823	0.2320	UP	0.0300
15q15.1	rs8042947	40033659	G	A	0.7233	6	0.2640	0.0480	1.30	3.71E-08	0.4360	BUB1B	0.3573	UP	0.1302	-0.5773	DOWN	0.0180
15q15.1	rs17722526	40028066	A	G	0.3494	6	0.3836	0.0414	1.47	1.94E-20	0.1115	RAD51	0.0710	UP	0.1519	0.2678	UP	0.0330
15q15.1	rs8026641	40046660	A	G	0.2774	6	0.4879	0.0426	1.63	2.28E-30	0.3138	RPUSD2	0.0658	UP	0.4069	0.2180	UP	0.0360
18q21.2	rs7232265	51289136	G	A	0.5140	6	0.2041	0.0404	1.23	4.28E-07	0.9095	MEX3C	-0.2279	**DOWN**	**5.75E-06**	-0.3942	DOWN	0.0190
18q21.2	rs8096627	51296370	C	A	0.4988	6	0.2134	0.0403	1.24	1.23E-07	0.8943	ME2	-0.2082	DOWN	0.0032	-0.2239	DOWN	0.0820
18q21.2	rs7232265	51289136	G	A	0.5140	6	0.2041	0.0404	1.23	4.28E-07	0.9095	MRO	-0.3032	DOWN	0.0034	-0.1191	DOWN	0.2600
20p11.22	rs911644	20985110	C	T	0.7829	6	0.2971	0.0531	1.35	2.20E-08	0.2556	RALGAPA2	-0.2148	DOWN	0.0098	-0.1700	DOWN	0.3900
20p11.22	rs2249048	21477034	A	C	0.6782	6	0.2944	0.0456	1.34	1.10E-10	0.8773	PAX1	0.1113	UP	0.0272	0.2582	UP	0.3100
20p11.22	rs6137387	21557266	C	T	0.8264	6	0.5215	0.0635	1.68	2.06E-16	0.7941	LOC284788	-0.0031	DOWN	0.4844	-0.0073	**DOWN**	**1.60E-05**

[a]Cytogenic region and genomic position based on NCBI genome build 38.

[b]Effect allele frequencty (EAF) is based on dbGAP ALFA project European population.

[c]The number of GWAS sets the GWAS meta-analysis result is based on.

Supplemental Table 11: Log2 Fold Change(FC) in gene expression from *EWSR1::ETS* knockdown (KD) among ESCLA cell lines. Genes 1 Mb up or downstream from lead variant were selected. Negative values display downregulation of gene expression under KD. Positive values display upregulation after KD. *P* value is for a one sample t.test. Only nominally significant (*P* value < 0.05) results in the 18 cell line set are displayed. Bold-face represents *P* values below Bonferroni-corrected threshold ($P < 2.42×10^{-4}$). For each region, genes are sorted by statistical significance in 18 cell line set.

Region	Gene	All 18 Cell Lines				5 cell lines with highest KD efficiency			
		Mean LOG2FC	MedianLOG2FC	SD LOG2FC	*P* value	Mean LOG2FC	Median LOG2FC	SD LOG2FC	*P* value
1p36.13	PAX7	-3.33	-2.87	1.40	**1.44E-08**	-3.96	-4.08	0.74	0.0003
	SDHB	-0.59	-0.61	0.39	**5.34E-06**	-0.80	-0.77	0.31	0.0046
	PADI2	-1.74	-1.60	1.29	**2.60E-05**	-2.02	-2.21	1.53	0.0414
	ALDH4A1	-0.83	-0.86	0.71	**1.13E-04**	-0.93	-1.25	0.64	0.0320
	KLHDC7A	-0.22	-0.20	0.22	5.52E-04	-0.25	-0.18	0.30	0.1320
	IGSF21	-1.10	-0.87	1.17	9.09E-04	-1.07	-0.73	1.34	0.1498
	MFAP2	0.83	0.85	0.88	9.28E-04	1.60	1.17	0.83	0.0125
	RCC2	-0.15	-0.09	0.28	0.0350	-0.25	-0.23	0.31	0.1444
1p36.22	EXOSC10	-0.53	-0.51	0.26	**1.00E-07**	-0.69	-0.61	0.25	0.0036
	SRM	-0.72	-0.57	0.55	**3.33E-05**	-0.50	-0.53	0.30	0.0199
	TARDBP	-0.34	-0.25	0.26	**3.58E-05**	-0.44	-0.26	0.43	0.0806
	DRAXIN	1.00	0.91	1.02	6.26E-04	0.69	1.20	0.84	0.1396
	PLOD1	0.72	0.51	0.77	0.0010	1.15	0.94	1.08	0.0753
	UBIAD1	-0.47	-0.54	0.51	0.0011	-0.44	-0.60	0.42	0.0796
	AGTRAP	-0.55	-0.56	0.63	0.0017	-0.85	-0.93	0.60	0.0333
	MTOR	-0.28	-0.27	0.37	0.0049	-0.52	-0.54	0.28	0.0146
	KIAA2013	-0.19	-0.16	0.26	0.0072	-0.21	-0.10	0.19	0.0669
	RBP7	-0.27	-0.25	0.37	0.0074	-0.08	-0.05	0.26	0.5276
	PEX14	-0.28	-0.25	0.42	0.0120	-0.25	-0.32	0.32	0.1589
	MAD2L2	-0.23	-0.15	0.43	0.0344	-0.57	-0.67	0.51	0.0662
5q32.3	PLAC8L1	-0.21	-0.19	0.20	4.00E-04	-0.30	-0.29	0.29	0.0768
	TCERG1	-0.30	-0.26	0.29	4.44E-04	-0.44	-0.37	0.38	0.0569
	GPR151	-0.23	-0.19	0.28	0.0023	-0.30	-0.19	0.38	0.1580
	PPP2R2B	-0.37	-0.21	0.50	0.0053	-0.42	-0.23	0.68	0.2381
	RBM27	-0.27	-0.22	0.48	0.0297	-0.68	-0.41	0.70	0.0959
6p22.1	GNL1	-0.41	-0.44	0.34	**7.36E-05**	-0.48	-0.49	0.32	0.0299
	GABBR1	0.35	0.27	0.36	7.14E-04	0.38	0.35	0.12	0.0019
	OR12D2	-0.18	-0.23	0.18	7.25E-04	-0.17	-0.22	0.15	0.0644
	HLA-A	0.57	0.43	0.80	0.0074	1.14	0.86	1.04	0.0707
	OR2H2	-0.14	-0.17	0.21	0.0085	-0.23	-0.35	0.23	0.0872
	RPP21	-0.32	-0.34	0.46	0.0090	-0.34	-0.52	0.44	0.1617
	PPP1R11	-0.24	-0.24	0.35	0.0101	-0.24	-0.28	0.34	0.1840
	HLA-F	0.31	0.32	0.48	0.0125	0.59	0.54	0.72	0.1443
	ZNF311	-0.18	-0.17	0.29	0.0153	-0.08	-0.15	0.17	0.3385
	OR14J1	-0.14	-0.09	0.23	0.0172	-0.12	-0.09	0.19	0.2115
	TRIM27	-0.15	-0.14	0.25	0.0195	-0.08	-0.14	0.13	0.2216
	HLA-L	0.39	0.39	0.72	0.0327	0.84	0.73	1.08	0.1559
	TRIM26	0.17	0.10	0.32	0.0415	0.21	0.11	0.23	0.1082
	OR12D3	-0.14	-0.10	0.27	0.0432	-0.19	-0.11	0.20	0.1052
6p25.1	SNRNP48	-0.42	-0.35	0.32	**3.58E-05**	-0.45	-0.36	0.43	0.0812
	RREB1	-0.43	-0.33	0.33	**4.10E-05**	-0.63	-0.60	0.34	0.0149
	BMP6	-0.77	-0.79	0.83	0.0011	-0.39	-0.69	0.88	0.3774
	CAGE1	-0.14	-0.10	0.18	0.0044	-0.17	-0.24	0.18	0.0934
	NRN1	-0.86	-0.75	1.32	0.0132	-1.64	-1.33	0.93	0.0166
	RIOK1	-0.35	-0.49	0.62	0.0278	-0.64	-0.84	0.51	0.0501
7q32.3	TMEM209	-0.43	-0.39	0.27	**3.74E-06**	-0.46	-0.39	0.24	0.0139
	ZC3HC1	-0.32	-0.27	0.33	6.23E-04	-0.46	-0.39	0.44	0.0826
	MKLN1	-0.25	-0.15	0.36	0.0076	-0.49	-0.34	0.50	0.0950
	SSMEM1	-0.11	-0.10	0.18	0.0150	-0.14	-0.08	0.12	0.0690
	KLHDC10	-0.21	-0.28	0.34	0.0187	-0.15	-0.23	0.36	0.3927
8q24.21	MYC	-0.48	-0.44	0.78	0.0180	-0.30	-0.61	0.73	0.4095
	GSDMC	-0.01	0.01	0.24	0.8629	-0.06	0.01	0.32	0.7200
10q21.2	ARID5B	2.02	1.87	0.87	**1.75E-08**	2.56	2.00	0.90	0.0032
	JMJD1C	0.72	0.62	0.49	**9.47E-06**	0.87	0.83	0.33	0.0043
	EGR2	-1.59	-1.57	1.71	0.0011	-1.18	-1.69	2.19	0.2948
	NRBF2	-0.33	-0.28	0.38	0.0020	-0.51	-0.56	0.24	0.0085
	RTKN2	-0.66	-0.38	0.91	0.0067	-0.75	-0.64	0.87	0.1251
	REEP3	0.32	0.10	0.55	0.0254	0.21	-0.14	0.69	0.5418
	MIR1296	0.21	0.13	0.37	0.0282	0.10	0.05	0.25	0.4303
	ADO	-0.27	-0.13	0.52	0.0410	-0.41	-0.40	0.62	0.2126
11q24.1	TECTA	0.32	0.20	0.40	0.0035	0.44	0.68	0.48	0.1146
	TBCEL	0.37	0.46	0.53	0.0084	0.40	0.45	0.69	0.2650
	UBASH3B	0.35	0.14	0.67	0.0405	0.69	0.47	1.11	0.2356
	GRIK4	0.31	0.16	0.61	0.0419	0.77	0.22	0.99	0.1580
12q14.3	LEMD3	0.21	0.15	0.23	0.0011	0.18	0.05	0.26	0.2040
	MSRB3	0.94	0.61	1.09	0.0019	1.24	-0.06	1.84	0.2055
	HMGA2	0.80	0.48	0.99	0.0033	1.09	0.88	1.34	0.1431
	LLPH	-0.21	-0.25	0.31	0.0107	-0.31	-0.40	0.19	0.0206
	GRIP1	0.55	0.45	0.84	0.0123	0.38	0.39	0.61	0.2344
	RPSAP52	0.13	0.11	0.21	0.0190	0.16	0.12	0.23	0.1951
14q13.3	SFTA3	-0.10	-0.10	0.17	0.0235	-0.14	-0.15	0.06	0.0056
	SLC25A21	-0.40	-0.48	0.69	0.0261	-0.80	-0.72	0.77	0.0822
15q15.1	KNSTRN	-0.88	-0.77	0.64	**1.92E-05**	-1.19	-1.07	0.92	0.0449
	INO80	-0.33	-0.30	0.24	**2.74E-05**	-0.49	-0.48	0.19	0.0042
	RAD51	-1.16	-0.98	0.92	**5.64E-05**	-1.41	-1.46	0.81	0.0174
	RMDN3	-0.37	-0.33	0.33	**1.93E-04**	-0.51	-0.35	0.39	0.0448
	BUB1B	-1.14	-0.87	1.09	3.47E-04	-1.31	-0.94	1.39	0.1025
	RHOV	-0.21	-0.18	0.23	0.0012	-0.25	-0.18	0.20	0.0488
	INAFM2	0.44	0.31	0.51	0.0017	0.14	0.09	0.22	0.2186
	RPUSD2	-0.32	-0.35	0.37	0.0022	-0.52	-0.44	0.20	0.0045
	THBS1	1.88	0.97	2.25	0.0025	2.54	0.79	3.18	0.1487
	PLCB2	0.33	0.21	0.40	0.0026	0.43	0.44	0.39	0.0701
	BAHD1	-0.16	-0.17	0.24	0.0102	-0.13	-0.22	0.18	0.1712
	EIF2AK4	0.24	0.19	0.36	0.0109	0.29	0.35	0.59	0.3352
	SRP14	0.14	0.10	0.21	0.0122	0.02	0.00	0.15	0.7486
	GPR176	0.66	0.52	1.00	0.0127	0.83	0.98	1.11	0.1706
	DNAJC17	-0.19	-0.20	0.29	0.0141	-0.24	-0.34	0.28	0.1205
	ZFYVE19	-0.21	-0.27	0.35	0.0197	-0.35	-0.60	0.37	0.0977
	IVD	0.21	0.19	0.41	0.0407	-0.01	0.00	0.39	0.9520
	VPS18	0.16	0.13	0.31	0.0427	0.14	0.21	0.38	0.4475
18q21.2	MRO	-0.95	-0.91	0.63	**6.60E-06**	-1.12	-0.92	0.29	0.0010
	MEX3C	0.49	0.48	0.41	**8.34E-05**	0.57	0.49	0.19	0.0024
	SKA1	-0.94	-0.80	0.84	**1.72E-04**	-0.83	-0.48	1.04	0.1511
20p11.22	NKX2-2	-0.87	-0.68	0.83	3.49E-04	-1.47	-0.70	1.29	0.0640
	RALGAPA2	0.56	0.54	0.56	5.66E-04	0.11	-0.08	0.47	0.6260
	XRN2	-0.15	-0.16	0.17	0.0018	-0.17	-0.08	0.18	0.0939

Supplemental Table 12. Summary of integrative analysis identifying potential etiological mechanisms and target genes for identified Ewing sarcoma susceptibility loci. Genes that reach statistical significance in Bonferroni-corrected threshold (or the top gene when none reached statistical significance) are listed for eQTL or *EWSR1-ETS* Knockdown expression. Nominally significant genes are displayed in grey font. The top gene or statistically significant genes in one method are displayed for the other method if the results were nominally significant.

Locus	GGAA enrichment	*FLI1* binding *enrichment*	GWAS risk allele on eQTL		*EWSR1-FLI* Knockdown Expression change		H3K27ac Hi-ChIP interaction between ≥ 4 GGAA mSat and gene promoter	CTCF Hi-ChIP interaction between ≥ 4 GGAA mSat and gene promoter
			eQTL genes	eQTL Effect	KD genes	KD effect		
1p36.22	X	X	*TARDBP*	DOWN	*TARDBP*	DOWN	X	-
			CASZ1	DOWN	-	-	-	-
			-	-	*SRM*	DOWN	-	X
			-	-	*EXOSC10*	DOWN	-	-
1p36.13	-	-	-	-	*PAX7*	DOWN	X	-
			-	-	*SDHB*	DOWN	-	-
			-	-	*PADI2*	DOWN	X	X
			-	-	*ALDH4A1*	DOWN	X	-
5q32.3	-	-	*RBM27*	DOWN	*RBM27*	DOWN	X	-
			PLAC8L1	DOWN	*PLAC8L1*	DOWN	-	-
6p25.1	X	X	*RREB1*	UP	*RREB1*	DOWN	-	-
			-	-	*SNRNP48*	DOWN	-	-
6p22.1	-	-	-	-	*GNL1*	DOWN	-	X
7q32.3	X	X	*CPA4*	DOWN	-	-	-	-
			-	-	*TMEM209*	DOWN	-	-
8q24.21	X	X	*MYC*	UP	*MYC*	DOWN	X	-
10q21.2	X	-	*ADO*	UP	*ADO*	DOWN	-	-
			EGR2	UP	*EGR2*	DOWN	-	-
			-	-	*ARID5B*	UP	X	-
			JMJD1C	DOWN	*JMJD1C*	UP	-	X
11q24.1	X	X	*SC5D*	UP	-	-	-	-
			-	-	*TECTA*	UP	-	-
12q14.3	X	X	*TMBIM4*	UP	-	-	-	-
			-	-	*LEMD3*	UP	-	-
14q13.3	X	X	*PAX9*	DOWN	-	-	-	X
			MIPOL1	DOWN	-	-	-	-
			SLC25A21	DOWN	*SLC25A21*	DOWN	-	-
			-		*SFTA3*	DOWN	-	-
15q15.1	-	X	*SRP14*	DOWN	*SRP14*	UP	-	-
			KNSTRN	UP	*KNSTRN*	DOWN	X	-
			-	-	*INO80*	DOWN	-	-
			RAD51	UP	*RAD51*	DOWN	-	-
			-	-	*RMDN3*	DOWN	-	-
18q21.2	-	X	*MEX3C*	DOWN	*MEX3C*	UP	-	-
			MRO	DOWN	*MRO*	DOWN	-	-
			-	-	*SKA1*	DOWN	-	-
20p11.22	X	X	*RALGAPA2*	DOWN	*RALGAPA2*	UP	-	-
			-	-	*NKX2-2*	DOWN	X	X
