# Supplementary material for: Genome-wide association study meta-analysis identifies susceptibility loci informing Ewing sarcoma etiology and potential mechanisms of risk
Source: medRxiv. 2026 Feb 9:2026.02.06.26345779. Preprint. [Version 1] doi: 10.64898/2026.02.06.26345779 (PMC12919121; doi:10.64898/2026.02.06.26345779)
Supplement: Supplement 2 [file media-2.pdf]

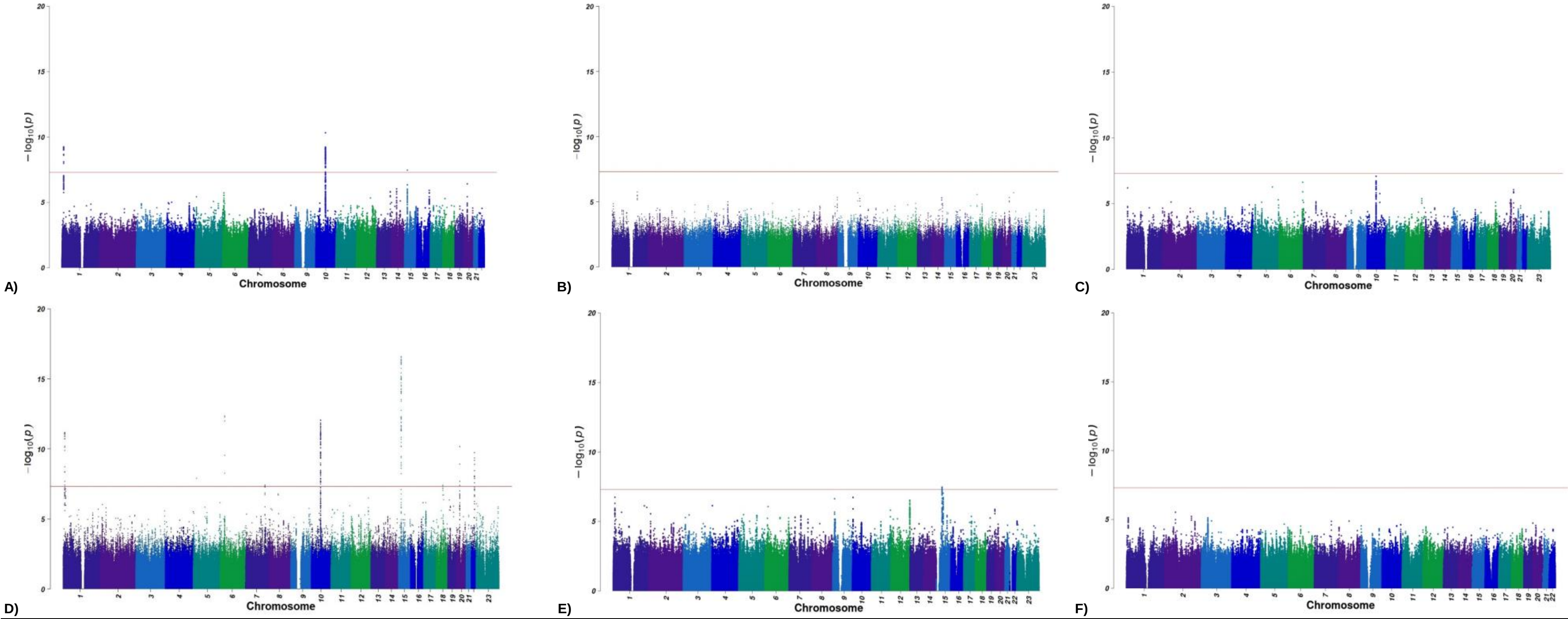

Supplemental Figure 1. Manhattan plots of -log10 *P* values for the association of each SNP with EwS risk in each GWAS set prior to meta-analysis. Association *P* values for each tested genetic variant are plotted. Chromosomes are plotted sequentially across the x-axis with the scale proportional to chromosomal size. Colors are used to visualize differences in chromosome. The red line indicates genome-wide significance (*P* <5×10-8). Variants with < 1% minor allele frequency were excluded from each study's GWAS. A) Institut Curie GWAS 1 set, B) Omni Set, C) CCSS, D) GSA Set, E) CESS, F) SJ Life Study. The estimated genomic inflation was lambdas ranged from 0.98 to 1.03.

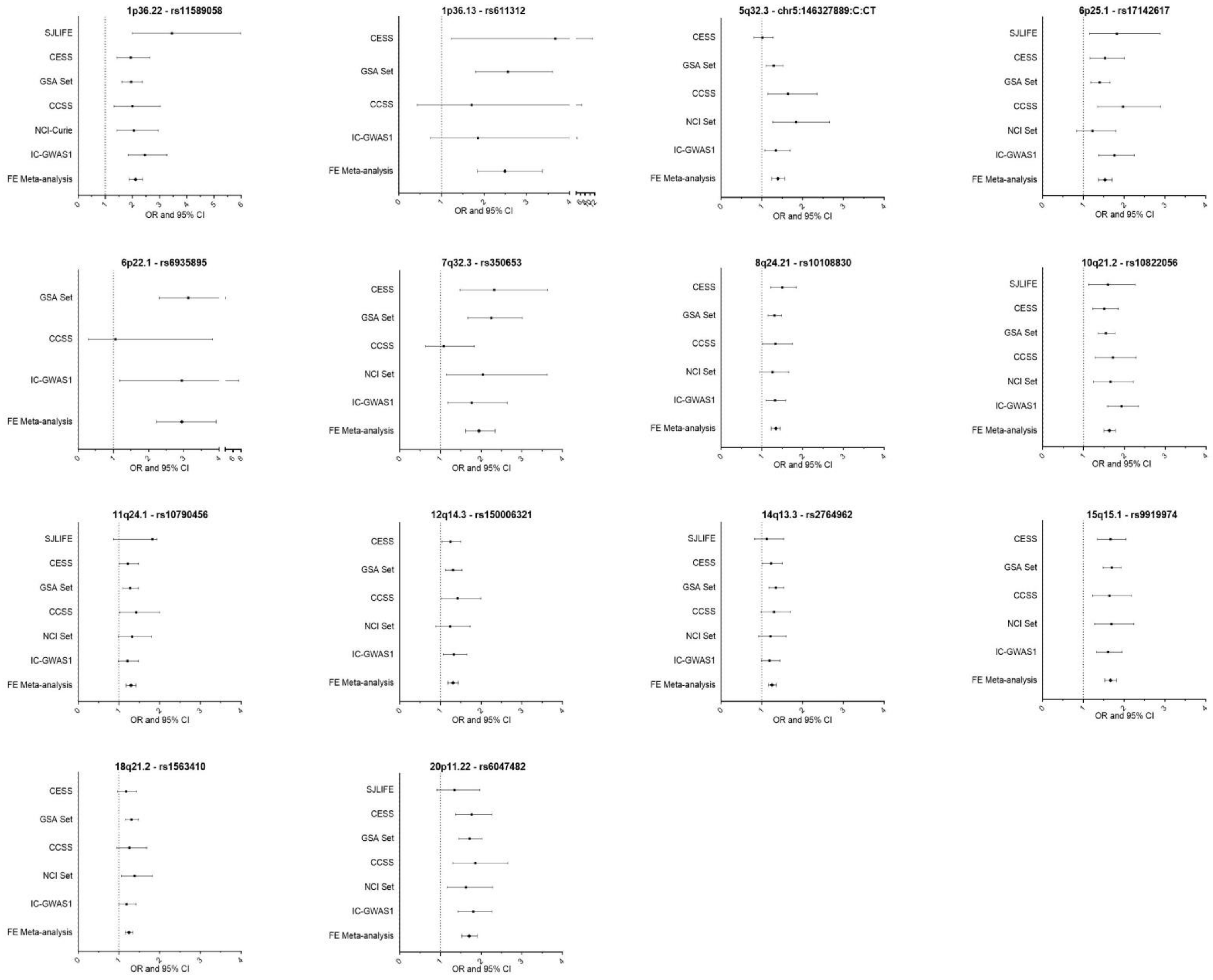

Supplemental Figure 2. Odds Ratio (OR) and 95% Confidence Interval (CI) for the lead variant for each genome wide significant locus in the Fixed Effects (FE) meta-analysis and across each study. Effects are not plotted when a lead variant was not present within the individual study.

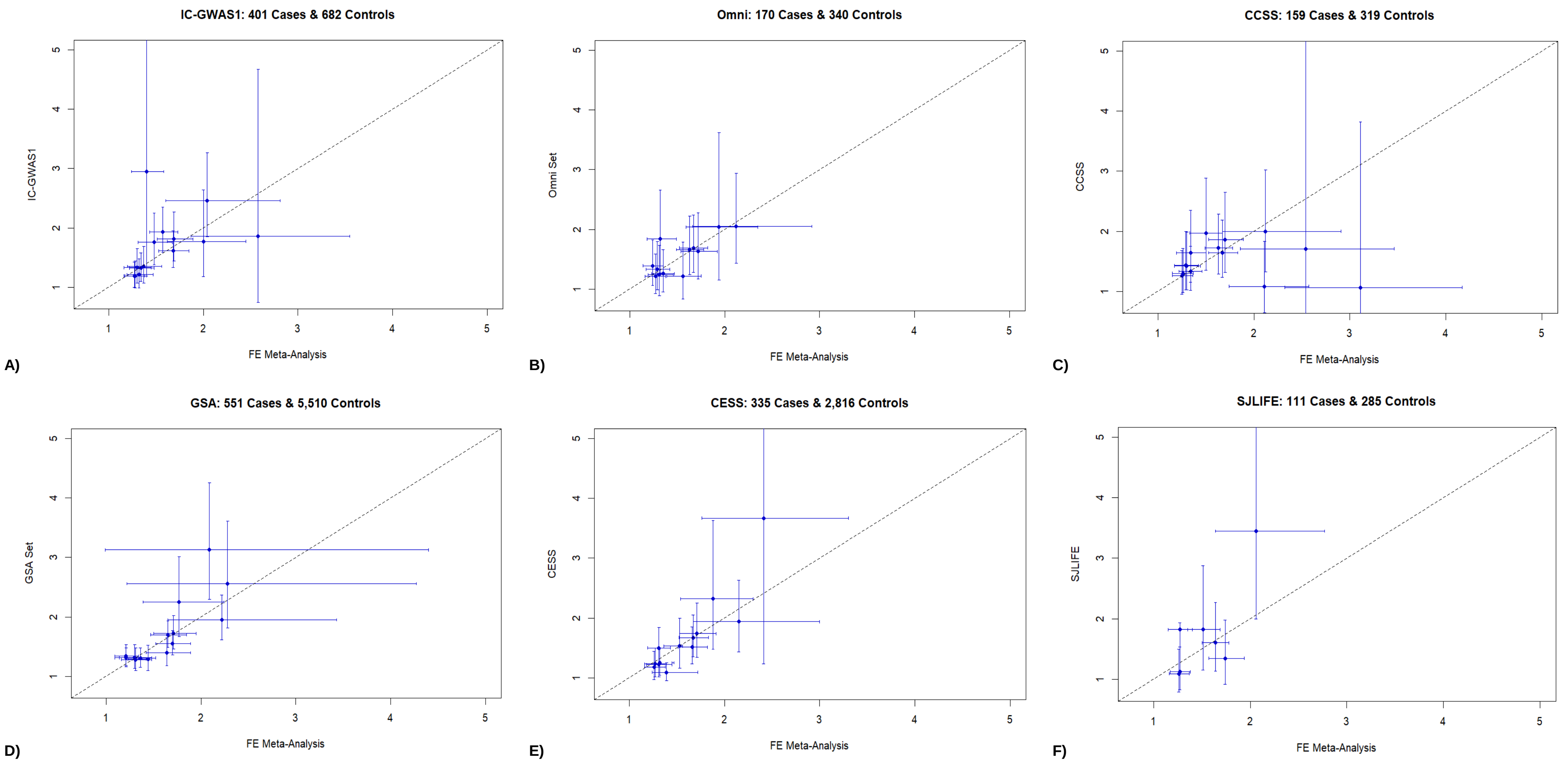

Supplemental Figure 3. Leave-one-out (LOO) comparison of Odds Ratios (OR) and 95% Confidence Intervals for Fixed Effects (FE) meta-analysis without each study and the study-specific effect. Each panel displays a different study and the FE meta-analysis without that study. Detailed results for variants in each study are provided in Supplemental Table 3. LOO meta-analysis results are given in **Supplemental Table 5**.

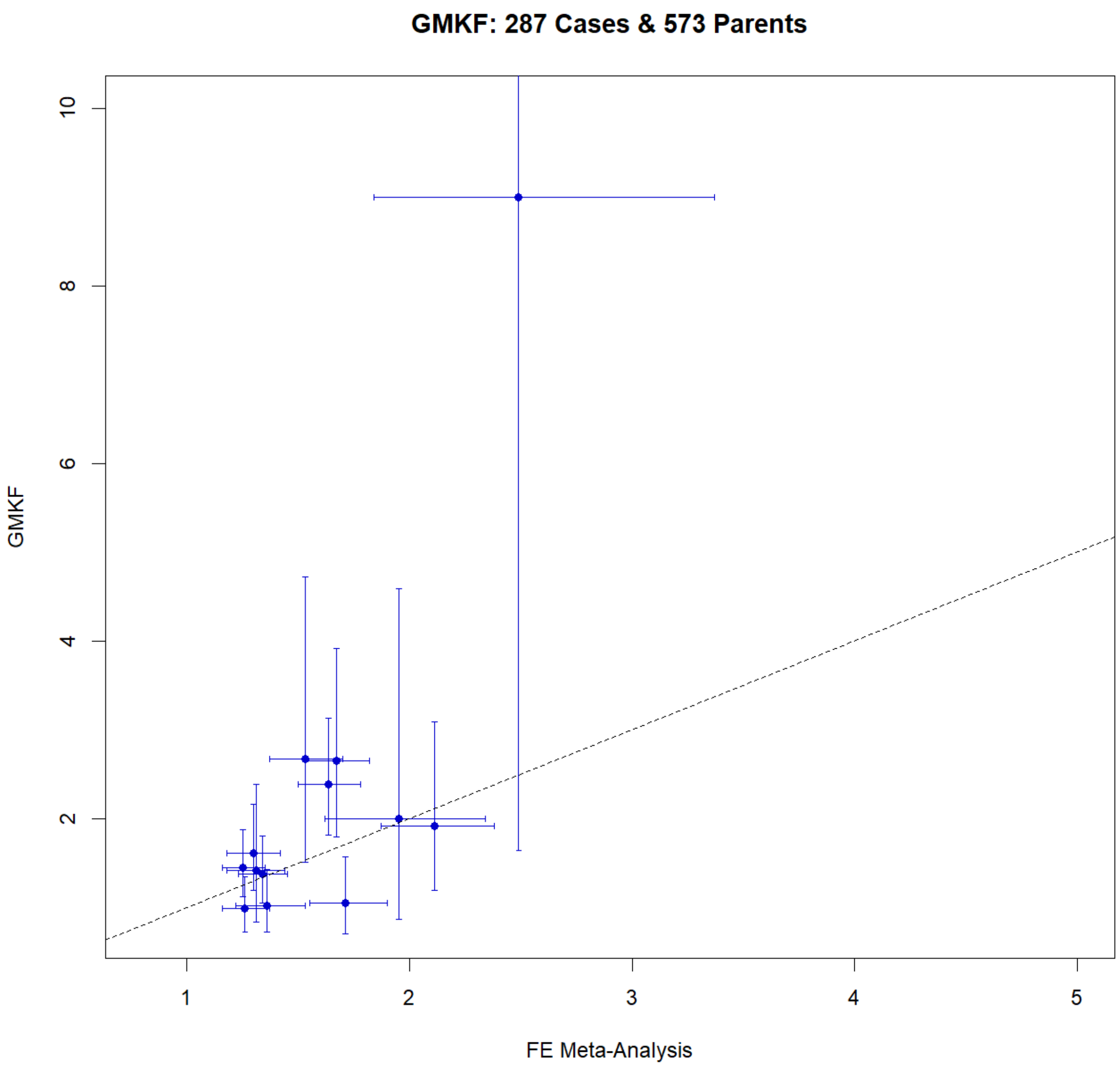

Supplemental Figure 4. The transmission disequilibrium test (TDT) odds ratio (OR) and 95% confidence interval (CI) in GMKF trio set for each variant in the main meta-analysis GWAS is plotted against the fixed effects meta-analysis OR and 95%CI. Detailed results for the variants in GMKF are given in **Supplemental Table 4**.

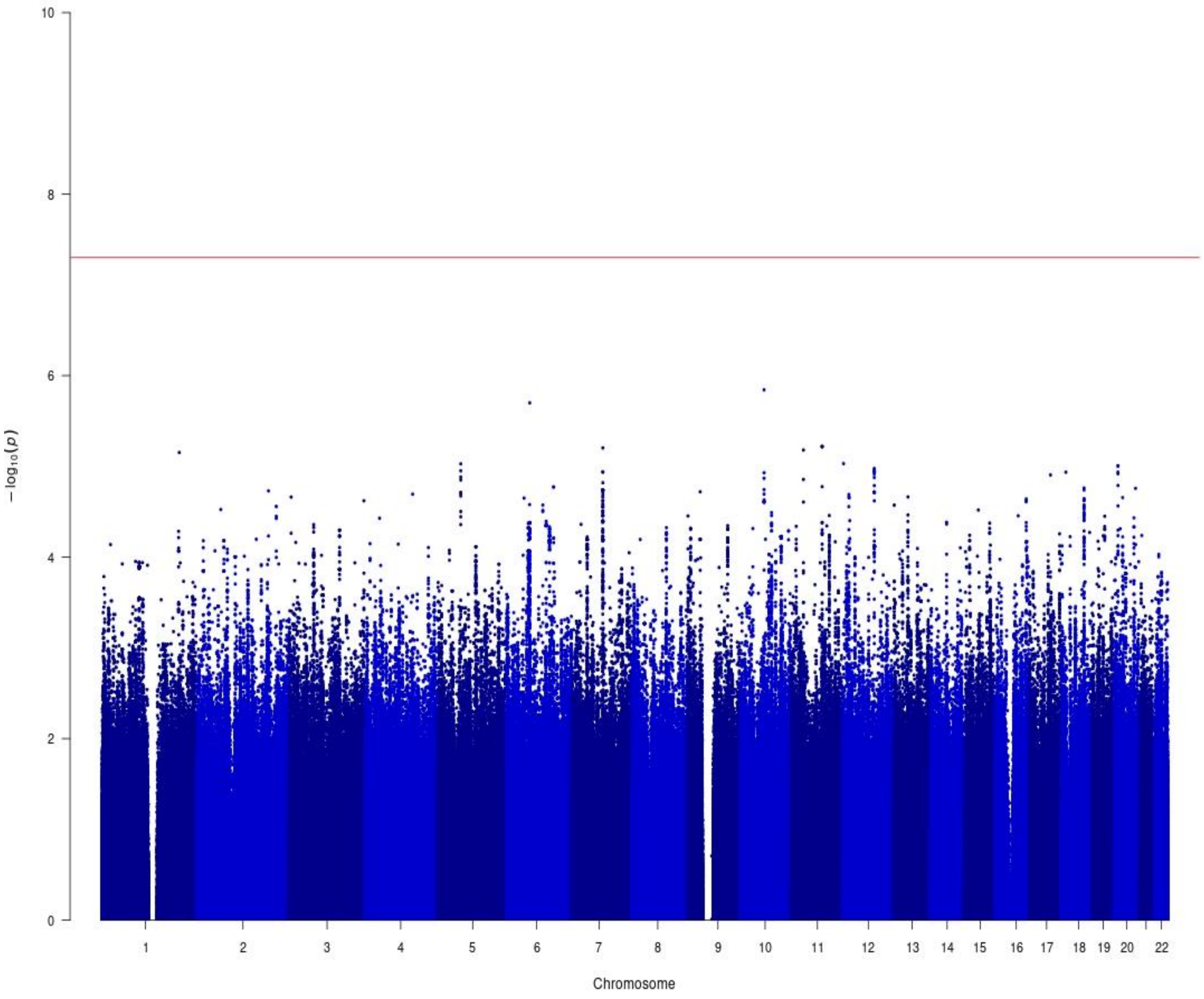

Supplemental Figure 5. Manhattan plot for fixed effects meta-analysis –log10 *P* values for the association of each SNP with sex among 709 males and 559 females with EwS. Association p-values for each tested genetic variant are plotted. Chromosomes are plotted sequentially across the x-axis with the scale proportional to chromosomal size. Colors are used to visualize differences in chromosome. The red line indicates genome-wide significance (*P*<5×10-8). Variants with < 1% minor allele frequency were excluded from each study's results prior to meta-analysis. Analysis included 8,839,636 SNPs. Variants with heterogeneity Q<0.05 were excluded from Manhattan plot. The estimated autosomal genomic inflation was lambda=0.96. P values for meta-analysis discovered loci are in **Table 2**. Phenotype distribution by study is provided in **Supplemental Table 8**.

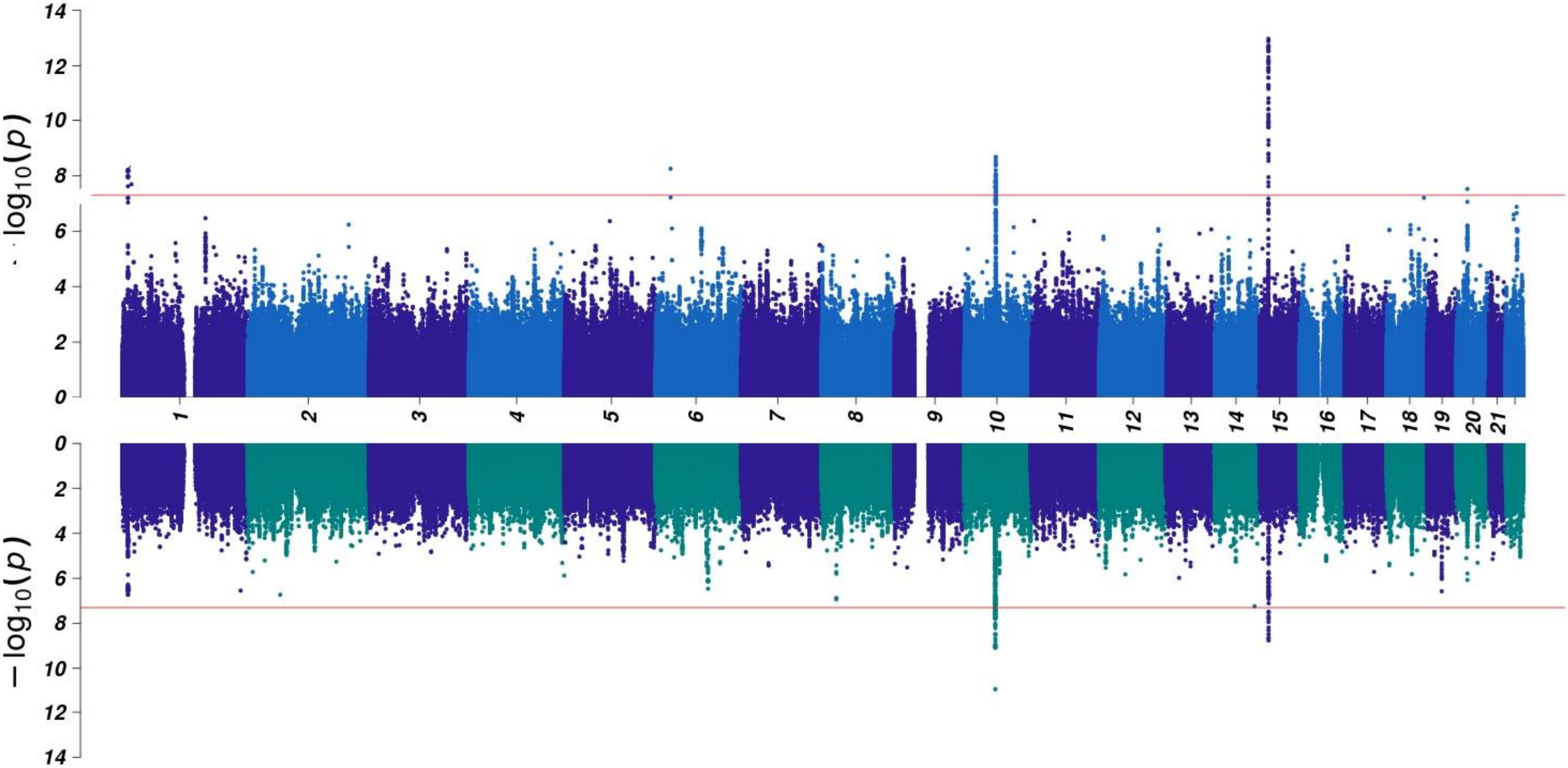

Supplemental Figure 6. Miami plot for fixed effects meta-analysis –log10 *P* values for the association of each SNP with EwS stratified by sex. Male EwS cases (N=378) and controls (N=2,618) are plotted on the top and female EwS cases (N=332) and controls (N=3,207) are plotted on the bottom. Association *P* values for each tested genetic variant are plotted. Chromosomes are plotted sequentially across the x-axis with the scale proportional to chromosomal size. Colors are used to visualize differences in chromosome. The red lines indicate genome-wide significance (*P* <5×10-8). Variants with < 1% minor allele frequency were excluded from each study's results prior to meta-analysis. Analysis included 8,443,513 variants in the male GWAS and 8,467,202 variants in the female GWAS. Variants with heterogeneity Q<0.05 were excluded from Manhattan plot. The estimated autosomal genomic inflation for lambda was 1.02 in the male GWAS and 1.00 in the female GWAS.

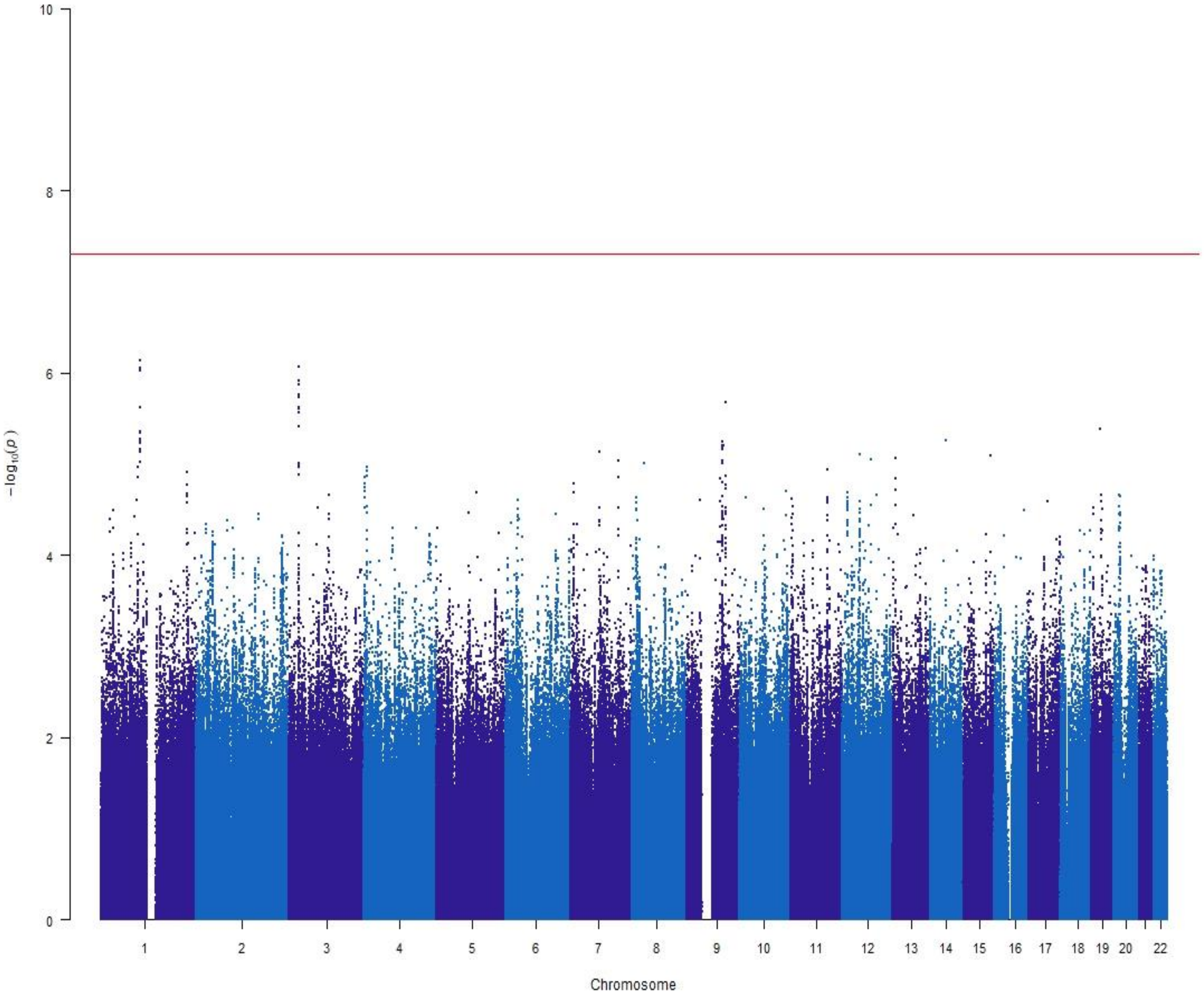

Supplemental Figure 7. Manhattan plot for fixed effects meta-analysis –log10 *P* values for the association of each SNP with metastasis at diagnosis (Yes/No) among 268 EwS cases with metastasis and 666 EwS cases without metastasis at diagnosis. Association *P* values for each tested genetic variant are plotted. Chromosomes are plotted sequentially across the x-axis with the scale proportional to chromosomal size. Colors are used to visualize differences in chromosome. The red line indicates genome-wide significance ($P<5\times10\text{-}8$). Variants with < 1% minor allele frequency were excluded from each study's results prior to meta-analysis. Variants with heterogeneity Q<0.05 were excluded from Manhattan plot. The autosomal genomic inflation factor was lambda=0.96. Analysis included 8,541,107 variants. Phenotype distribution by study is provided in **Supplemental Table 8**.

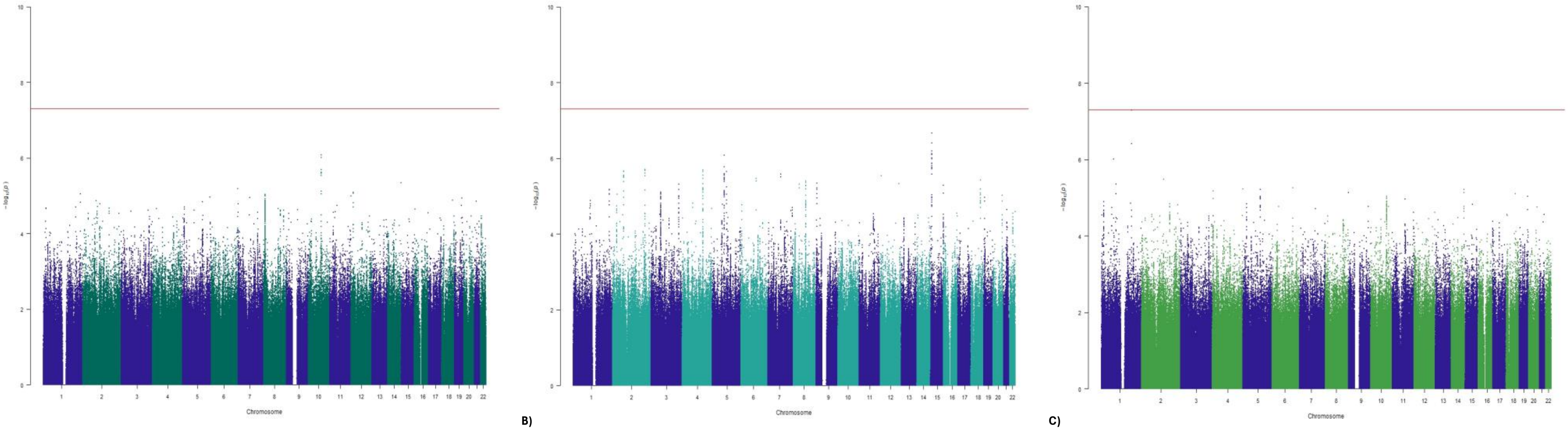

A) **B)** **C)**

Supplemental Figure 8. Manhattan plots for fixed effects meta-analysis –log10 *P* values for the association of each SNP with age at diagnosis. Association p-values for each tested genetic variant are plotted. Chromosomes are plotted sequentially across the x-axis with the scale proportional to chromosomal size. Colors are used to visualize differences in chromosome. The red line indicates genome-wide significance ($P<5\times10^{-8}$). Variants with < 1% minor allele frequencies were excluded from each study's results prior to meta-analysis. Variants with heterogeneity Q<0.05 were excluded from Manhattan plot. Three age phenotypes were evaluated **A)** young children (<10) vs. teens and adults (≥10), **B)** before puberty completion (≤15) vs. after puberty (>15), and **C)** pediatric onset (<20) vs. adult onset (≥20). Analysis included 8,755,664, 8,824,193, and 8,657,897 variants in phenotypes A-C respectively. Genomic inflation lambda=0.96-0.99. Phenotype distribution by study is provided in **Supplemental Table 8**.
