# Supplementary material for: Genome-wide association study meta-analysis identifies susceptibility loci informing Ewing sarcoma etiology and potential mechanisms of risk
Source: medRxiv. 2026 Feb 9:2026.02.06.26345779. Preprint. [Version 1] doi: 10.64898/2026.02.06.26345779 (PMC12919121; doi:10.64898/2026.02.06.26345779)
Supplement: Supplement 3 [file media-3.docx]

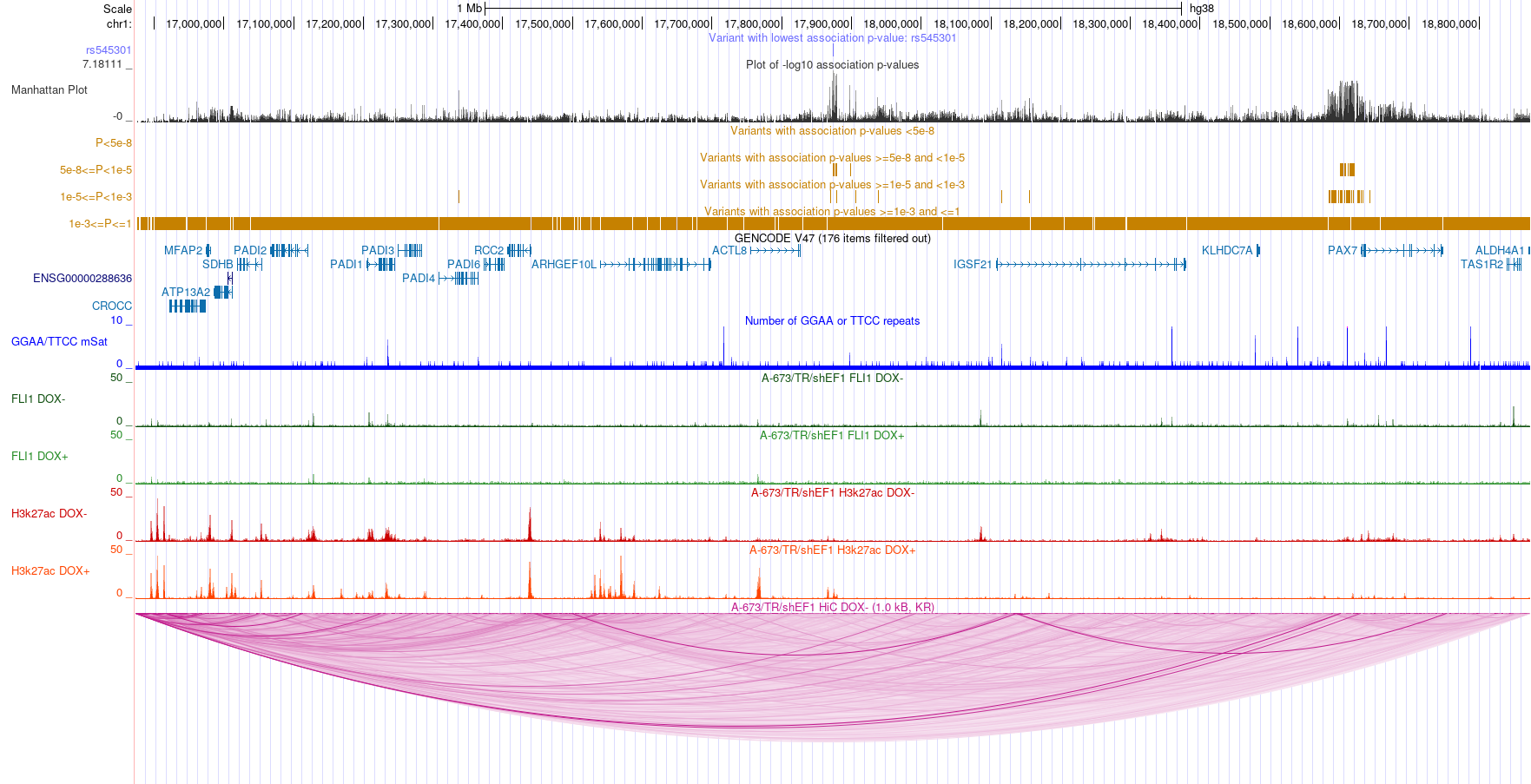


**Extended Data Figure 1: 1p36.13 Locus**. UCSC Genome browser view of meta-analysis results and publicly available data from the EwS cell line atlas. Plot displays meta-analysis results, genes 1 Mb up and downstream of the lead variant, GGAA-repeats, FLI1 binding, H3K27ac, and HiC loops.


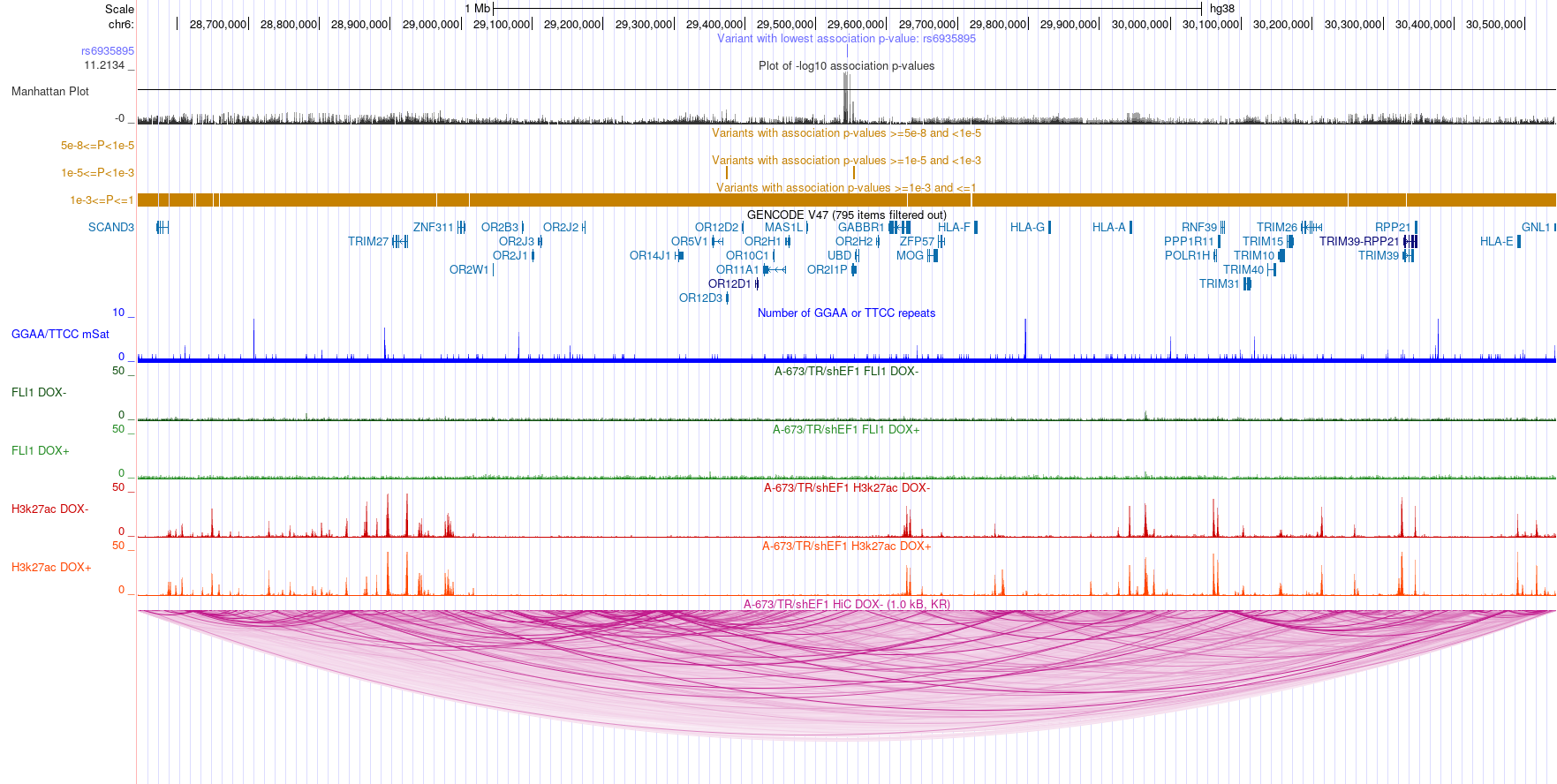


**Extended Data Figure 2: 6p22.1 Locus**. UCSC Genome browser view of meta-analysis results and publicly available data from the EwS cell line atlas. Plot displays meta-analysis results, genes 1 Mb up and downstream of the lead variant, GGAA-repeats, FLI1 binding, H3K27ac, and HiC loops.


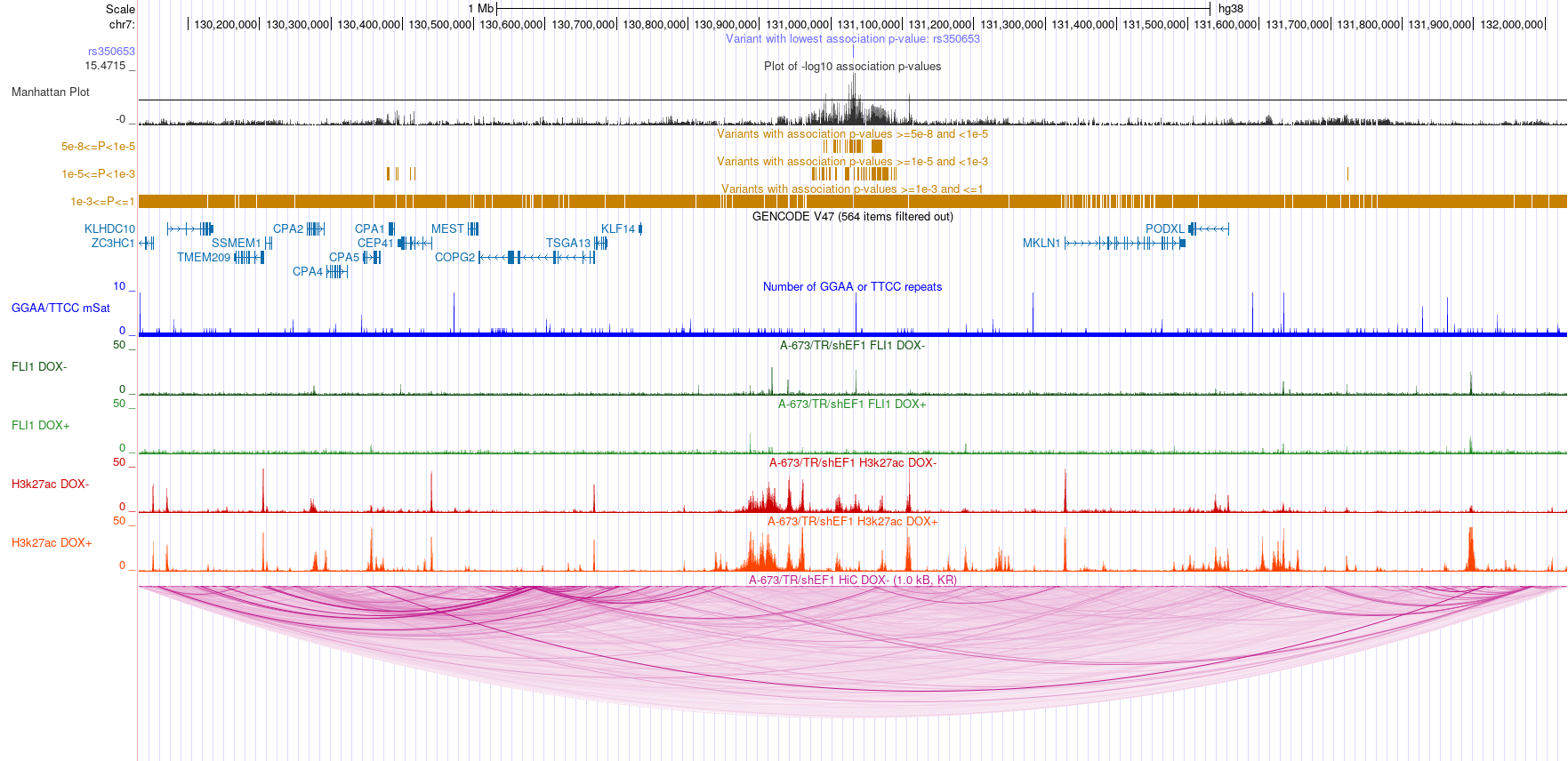


**Extended Data Figure 3: 7q32.3 Locus**. UCSC Genome browser view of meta-analysis results and publicly available data from the EwS cell line atlas. Plot displays meta-analysis results, genes 1 Mb up and downstream of the lead variant, GGAA-repeats, FLI1 binding, H3K27ac, and HiC loops.


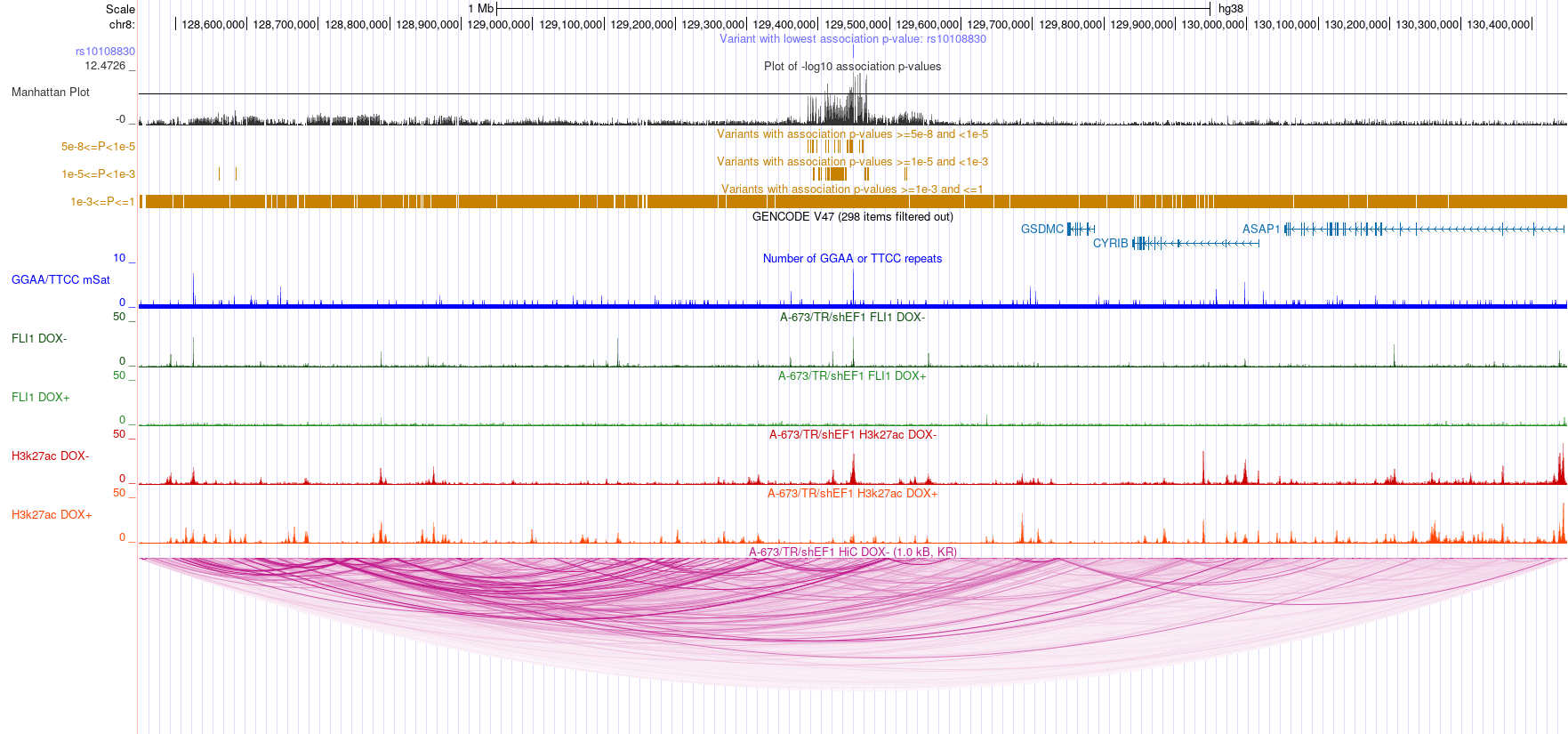


**Extended Data Figure 4: 8q24.21 Locus**. UCSC Genome browser view of meta-analysis results and publicly available data from the EwS cell line atlas. Plot displays meta-analysis results, genes 1 Mb up and downstream of the lead variant, GGAA-repeats, FLI1 binding, H3K27ac, and HiC loops.


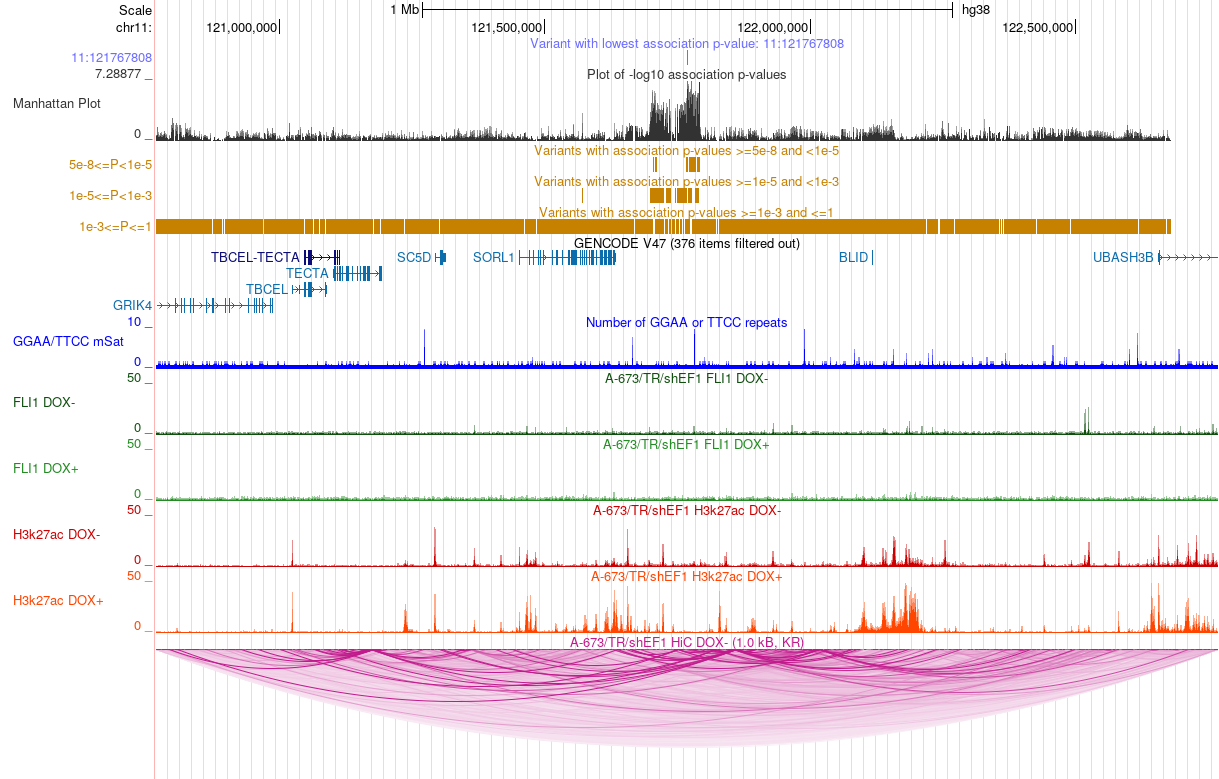


**Extended Data Figure 5: 11q24.1 Locus**. UCSC Genome browser view of meta-analysis results and publicly available data from the EwS cell line atlas. Plot displays meta-analysis results, genes 1 Mb up and downstream of the lead variant, GGAA-repeats, FLI1 binding, H3K27ac, and HiC loops.


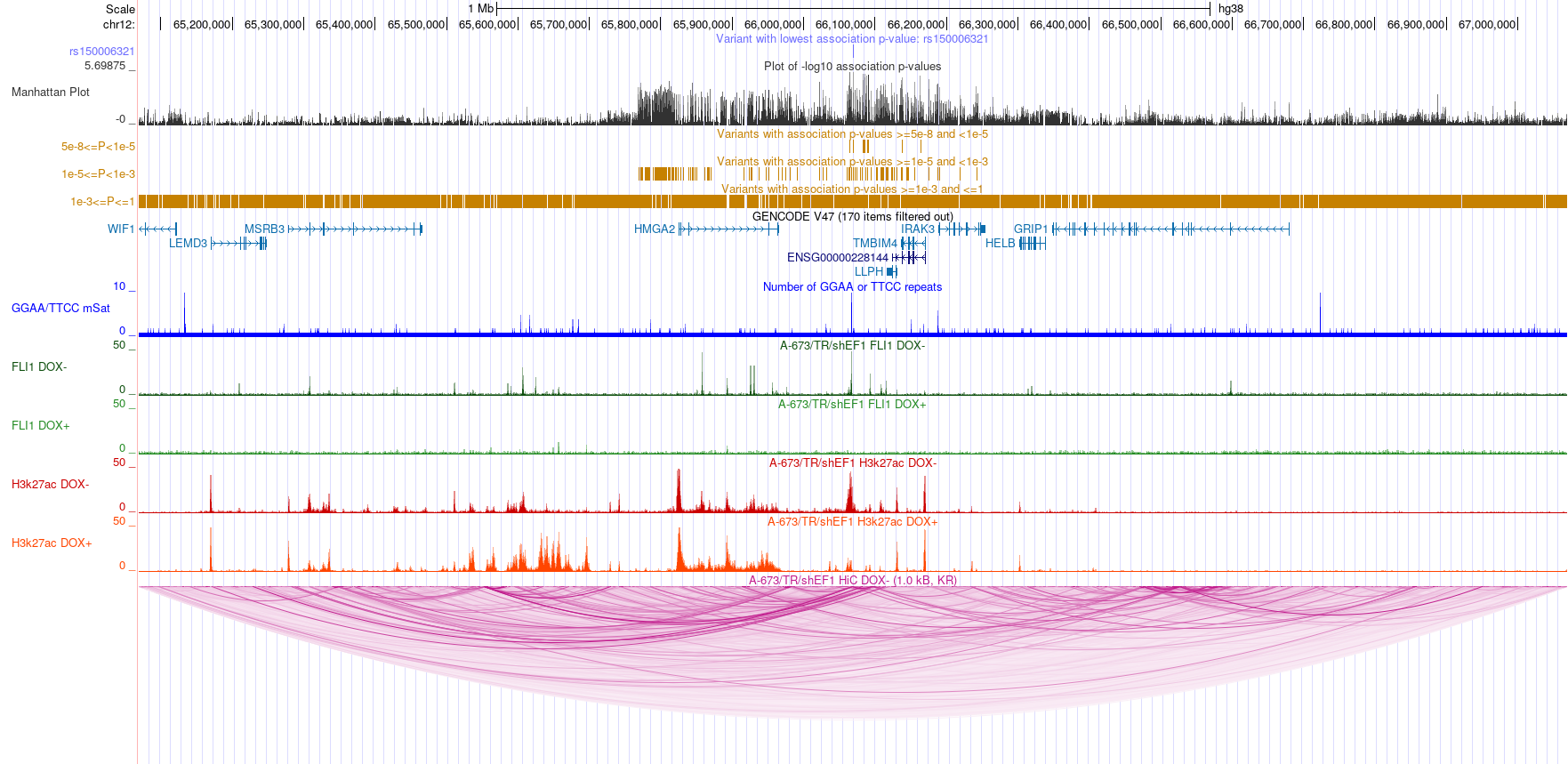


**Extended Data Figure 6: 12q14.3 Locus**. UCSC Genome browser view of meta-analysis results and publicly available data from the EwS cell line atlas. Plot displays meta-analysis results, genes 1 Mb up and downstream of the lead variant, GGAA-repeats, FLI1 binding, H3K27ac, and HiC loops.

**
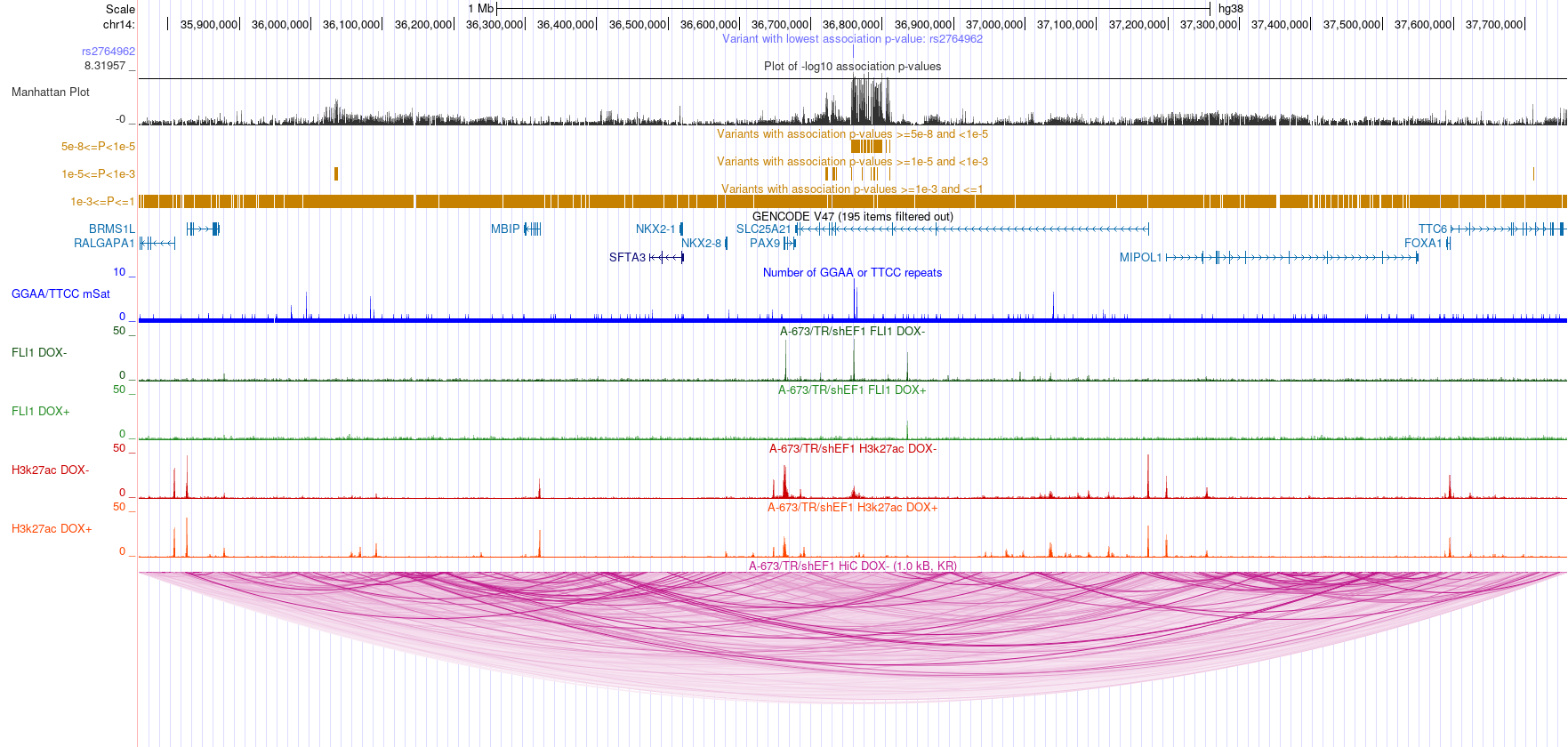
 Extended Data Figure 7: 14q13.3 Locus**. UCSC Genome browser view of meta-analysis results and publicly available data from the EwS cell line atlas. Plot displays meta-analysis results, genes 1 Mb up and downstream of the lead variant, GGAA-repeats, FLI1 binding, H3K27ac, and HiC loops.

**
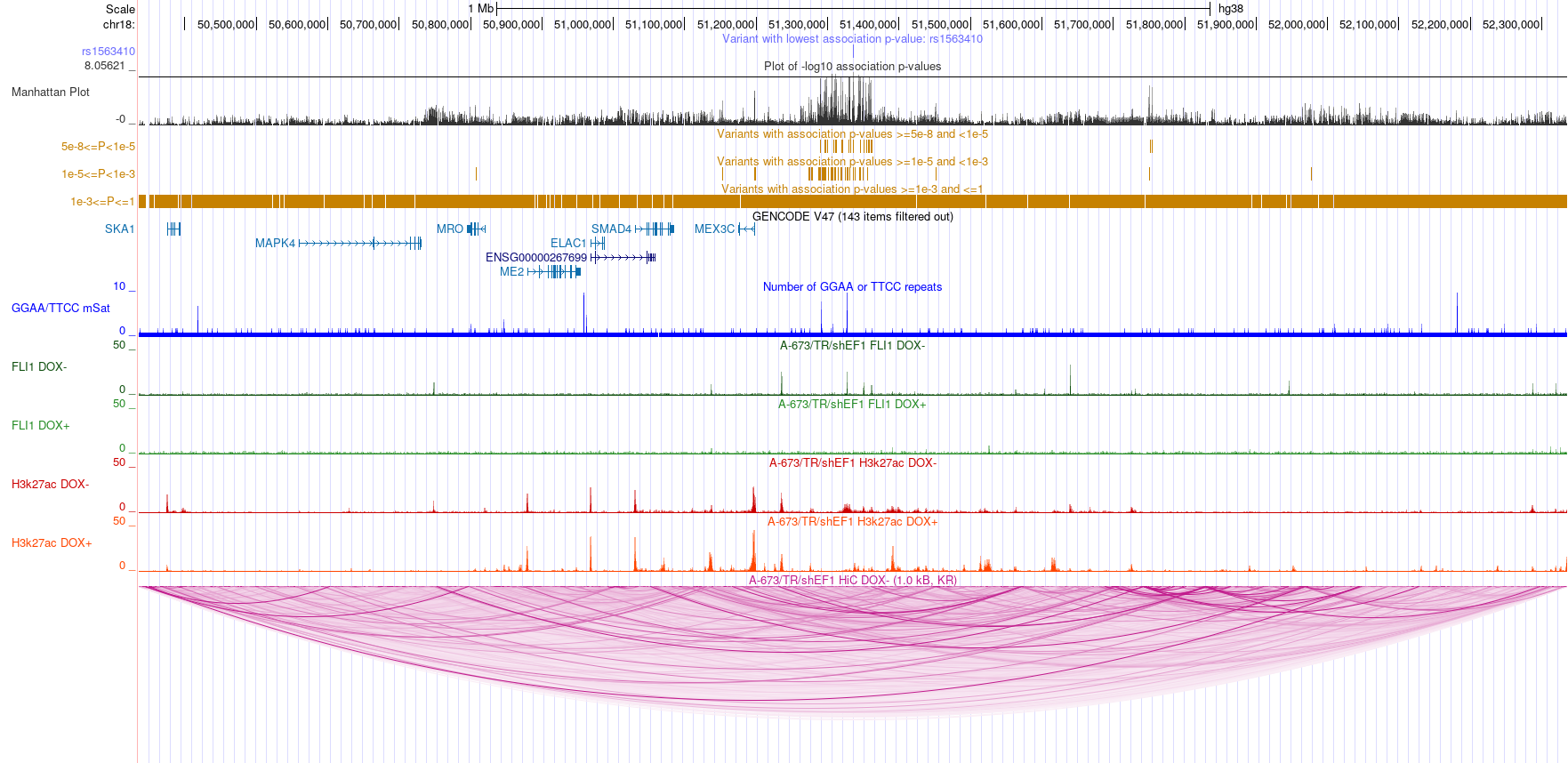
 Extended Data Figure 8: 18q21.1 Locus**. UCSC Genome browser view of meta-analysis results and publicly available data from the EwS cell line atlas. Plot displays meta-analysis results, genes 1 Mb up and downstream of the lead variant, GGAA-repeats, FLI1 binding, H3K27ac, and HiC loops.
